# Integrated Analysis of Widely Targeted Metabolomics and Transcriptomics Reveals the Effects of Transcription Factor NOR-like1 on Alkaloids, Phenolic Acids, and Flavonoids in Tomato at Different Ripening Stages

**DOI:** 10.3390/metabo12121296

**Published:** 2022-12-19

**Authors:** Xinyu Yang, Xiaodan Zhao, Daqi Fu, Ying Zhao

**Affiliations:** 1 School of Food and Health, Beijing Technology and Business University, Beijing 100048, China; 2 Laboratory of Fruit Biology, College of Food Science & Nutritional Engineering, China Agricultural University, Beijing 100083, China

**Keywords:** tomato, widely targeted metabolomics, transcriptomics, NOR-like1, alkaloids, phenolic acids, flavonoids

## Abstract

Tomato is abundant in alkaloids, phenolic acids, and flavonoids; however, the effect of transcription factor NOR-like1 on these metabolites in tomato is unclear. We used a combination of widely targeted metabolomics and transcriptomics to analyze wild-type tomatoes and CR-NOR-like1 tomatoes. A total of 83 alkaloids, 85 phenolic acids, and 96 flavonoids were detected with significant changes. Combined with a KEGG enrichment analysis, we revealed 16 differentially expressed genes (DEGs) in alkaloid-related arginine and proline metabolism, 60 DEGs were identified in the phenolic acid-related phenylpropane biosynthesis, and 30 DEGs were identified in the flavonoid-related biosynthesis pathway. In addition, some highly correlated differential-expression genes with differential metabolites were further identified by correlation analysis. The present research provides a preliminary view of the effects of NOR-like1 transcription factor on alkaloid, phenolic acid, and flavonoid accumulation in tomatoes at different ripening stages based on widely targeted metabolomics and transcriptomics in plants, laying the foundation for extending fruit longevity and shelf life as well as cultivating stress-resistant plants.

## 1. Introduction

Tomato (*Solanum lycopersicon*) is the world’s most valuable fruit and vegetable crop, and it is rich in phenolic compounds (phenolic acids and flavonoids) and glycoside alkaloids (tomatine) [1,2,3,4]. Alkaloids are preventive secondary metabolites present in plant tissues. Steroid glycoside alkaloids (SGAs) are nitrogenous secondary metabolites primarily identified in the Solanaceae species. SGAs protect plants from insects, bacteria, and viruses that serve essential roles in defending against biological and non-biological stresses [5,6,7]. Alkaloids in wild-type tomatoes exist at high levels during early developmental stages and decrease gradually at maturity [8]. Phenolic acids in tomatoes are dominated by hydroxycinnamic acid and its conjugates; chlorogenic acid and caffeic acid present in tomatoes are the most extensively investigated [9,10]. Phenolic acids are effective as components of the plant defense system against UV, insects, viruses, and bacteria [11,12], as well as having a remarkable effect on color retention, retarding microbial development, and extending shelf life [13]. There are over 500 different flavonoids in tomatoes, which are mainly categorized into flavones, flavonols, flavanones, flavanols, proanthocyanidins, and isoflavones, depending on their glycosidic structures [14,15]. Naringenin chalcone is among the major flavonoids, as well as various glycoconjugates of quercetin and kaempferol [16]. Flavonoids have excellent antioxidant and anti-inflammatory characteristics, and fruit ripening in tomatoes is related to flavonoid accumulation [17].

Tomatoes undergo sharp changes in metabolism during the development of their fruit, and the metabolite content determines their nutritional value [15,18,19], making it an outstanding model for the study of maturation and secondary metabolism pathways in fleshy fruits [20]. The ripening of fruit is a sophisticated biogenetic process controlled by elements such as hormones, environmental signals, and transcription factors (TFs) and involves drastic variations in chemical composition, colors, textures, and flavors, as well as other sensory characteristics that directly influence the shelf life and quality of fruits [21,22,23,24]. Molecular genetic investigations indicate that the ripening of tomato fruit is controlled by sequences of ripening-related TFs and an ethylene-coordinated transcriptional regulatory network [25,26]. A total of 2026 genes have been identified as TFs in tomatoes, of which 516 have been linked to fruit ripening [27]. The various natural maturation inhibitory mutants in tomatoes have already been used to examine fruit shelf-life extension—for instance ripening inhibitor (rin), non-ripening (nor), colorless non-ripening (cnr), and never ripening (nr) [4,23]. There are 101 NAC TFs in the tomato genome and the majority of NAC proteins include a fully functional N-terminal DNA-binding structural region that is well conserved, as well as a variable C-terminal structural domain [23,24]. NAC1 [28,29], NAC4 [30], NAC9 [31], and NOR-like1 [24] have been shown previously to be involved in tomato-ripening regulation. It has been demonstrated that knock-out of NOR-like1 delays the start of fruit ripening by 14 days, reduces ethylene production, slows down softness and chlorophyll loss, and decreases the accumulation of lycopene [24]. However, the effect of NOR-like1 on tomato metabolites is still unclear. In this study, to reveal the effect of NOR-like1 on the metabolites of tomato at green ripening (GR), 3 days after the color break (BR+3) and 9 days after the color break (BR+9) we combined widely targeted metabolomics with transcriptomics to screen the three most distinctly different metabolites (alkaloids, phenolic acids, and flavonoids) and the associated DEGs, with KEGG-pathway enrichment analyses. In addition, relevant pathways of metabolism were further analyzed, and correlation network analyses were performed for different metabolites and different expression genes to provide deeper insights into the effects of NOR-like1 on alkaloids, phenolic acids, and flavonoids during tomato maturation. The present study may contribute to further investigation of the effect of NOR-like1 on metabolites in tomatoes at the various stages of maturation and could help to enhance tomato quality as well as extend the preservation period.

## 2. Materials and Methods

### 2.1. Plant Materials and Sample Preparation

Cultivated wild-type tomato Ailsa Craig (AC) and a NOR-like1 tomato transgenic line employing CRISPR/Cas9 gene-editing techniques were both grown in a greenhouse at China Agricultural University. A total of 18 samples of wild-type and CR-NOR-like1 fruits were collected and sampled at the green-ripening (GR) stage 3 days after the color break (BR+3) and 9 days after the color break (BR+9), including three biological replicates per period, and each sample was derived from six fruits, which were immediately frozen after sampling in liquid nitrogen at −80 °C and preserved until use. The biological samples were freeze-dried with a vacuum freeze dryer (Scientz-100F). Using a mixer mill with zirconia beads (MM 400, Retsch, Hamburg, Germany), the freeze-dried samples were pulverized at 30 Hz for 1.5 min. A total of 100 mg of the lyophilized powders were added by dissolving them in 1.2 mL 70% of the methanol solution, mixing by vortex for 30 s six times every 30 min, and leaving the samples at 4 °C in the fridge overnight. Before UPLC-MS/MS analysis, the extracts were filtered after centrifugation for 10 min on a 12,000 rpm centrifuge (SCAA-104, pore size 0.22 μm; ANPEL, Shanghai, China).

### 2.2. Widely Targeted Metabolic Analysis

Metabolite analysis was performed using UPLC (SHIMADZU Nexera X2) and tandem mass spectrometry (MS/MS, Applied Biosystems 4500 QTRAP) for metabolite analyses. Chromatographic separation was performed on a column (Agilent SB-C18, 1.8 µm, 2.1 mm × 100 mm), mobile phase-A in deionized water (containing 0.1% (*v*/*v*) formic acid), and phase-B in acetonitrile (containing 0.1% (*v*/*v*) formic acid). The gradient of elution was as follows: the B-phase was increased from 5% at 0 min up to 95% at 9.00 min and remained at 95% until 10 min, then the B-phase percentage was reduced to 5% at 10.00–11.15 min and equilibrated to 5% until 14 min. The flow rates were controlled with 0.35 mL/min, 40 °C column temperature, and 4 μL injection volume. Flow-throughs were alternately linked to the ESI-triple quadrupole linear ion trap (QTRAP)-MS.

LIT and triple quadrupole (QQQ) were obtained on a triple quadrupole linear-ion-trap mass spectrometer (QTRAP) with a UPLC/MS/MS system operating in the positive-ion mode (ESI+) and the negative-ion mode (ESI−). Operation of the ESI source parameters were as follows: ion source and turbo spray; source temperature of 550 °C; ion spray voltage of (IS) 5.5 kV (ESI+)/−4.5 kV (ESI−); ion-source gas I (GSI) of 50 psi, gas II (GSII) of 60 psi, and curtain gas (CUR) of 25.0 psi; and collision-activated dissociation (CAD) set to high. The tuning and mass calibration of the instrument was carried out in QQQ and LIT mode with 10 and 100 μmol/L of polypropylene glycol solution, respectively. QQQ scans were carried out in the MRM mode with the collision medium gas (nitrogen) setting. Further optimization of DP and CE was carried out for single MRM ion pairs. The set of specific pairs of MRM ions was monitored during each period according to the number of metabolites eluted during each period.

### 2.3. Differential-Metabolite (DEM) Analysis

Pre-processing of the data was performed and the data were analyzed by multivariate statistical analysis, which included principal component analysis (PCA) and orthogonal partial-least-squares discriminant analysis (OPLS-DA). A powerful tool to identify global patterns in multivariate experimental data, PCA provides a preliminary insight into the variability of overall metabolism among samples and the magnitude within groups of samples. OPLS-DA can filter messages in metabolites that are not correlated with categorical variables, thereby accurately analyzing inter-group differences in metabolites, which could further improve the resolving power and effectiveness of the model. Metabolite identification was conducted by matching the mass spectrum to the reference library MetWare database (MWDB). MWDB was constructed based on the standard compounds or public database like METLIN. Variable weight value (VIP) and *p*-values in t-tests were used to filter significantly different metabolites during different periods of growth between wild-type and CR-NOR-like1 tomatoes, and FC > 2 or FC < 0.5, along with *p* < 0.053 and VIP > 1, was satisfied to identify the differential compounds. KEGG annotation of significant DEM- and KEGG-related pathway analyses were conducted, resulting in the identification of critical pathways with the highest differential correlation to DEMs.

### 2.4. Transcriptomic Analysis

Using the procedure according to the RNeasy Mini Kit (Qiagen, Hilden, Germany), we isolated total RNA from the fruits and digested them with DNaseI (Qiagen, Germany) for genomic DNA removal. RNA was checked for purity with a NanoPhotometer^®^ spectrophotometer (IMPLEN, Westlake Village, CA, USA). RNA-concentration measurement was performed in a Qubit^®^ RNA Assay Kit in the Qubit^®^ 2.0 Flurometer (Life Technologies, Carlsbad, CA, USA). Detection of degradation and decontamination of RNA was carried out with a 1% agarose gel. Assessment of RNA integrity was carried out with the Bioanalyzer 2100 system’s RNA Nano 6000 assay kit (Agilent Technologies, Santa Clara, CA, USA). The RNA library contained total RNA, ≥1 ug. After the cDNA libraries were constructed, the libraries were tested for quality. After the library detection was qualified, the various libraries were pooled based on the valid concentration and the amount of target downstream data required for Illumina sequencing generated paired-end reads of 150 bp. The preliminary quality of the raw sequence data from the sequencer was analyzed to obtain raw sequencing data. Clean data were obtained by removing all low-quality sequences and the subsequent analysis was based on the clean reads. The reference genome and its annotation files were downloaded from the indicated websites and indexed by using HISAT v2.1.0 to compare the clean reads with the reference genome.

### 2.5. Quantification of Gene-Expression Levels

Gene alignments were calculated using featureCounts v1.6.2, followed by the FPKM of each gene according to its length. FPKM is currently used as the most common method available to assess gene-expression levels.

### 2.6. Differential Analysis and Differential Gene-Enrichment Analysis

Differentials expressed among the two groups were analyzed with DESeq2 v1.22.1 and corrected for *p*-values to obtain the false-discovery rate (FDR) using the Benjamini and Hochberg method. |log_2_ foldchange| and FDR were employed as significantly differentially expressed thresholds. KEGG is a test for hypergeometry and path cell-based hypergeometric distribution based on enrichment analysis.

## 3. Results

### 3.1. Widely Targeted Metabolomic Differential Analysis

**Figure 1:**
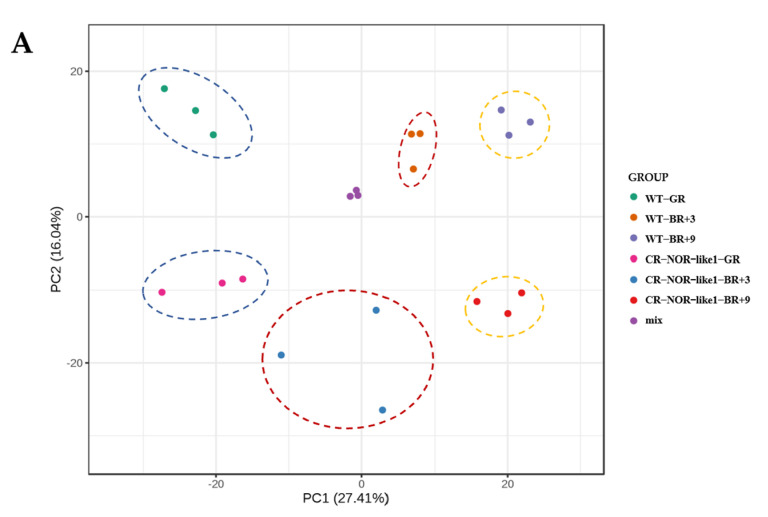

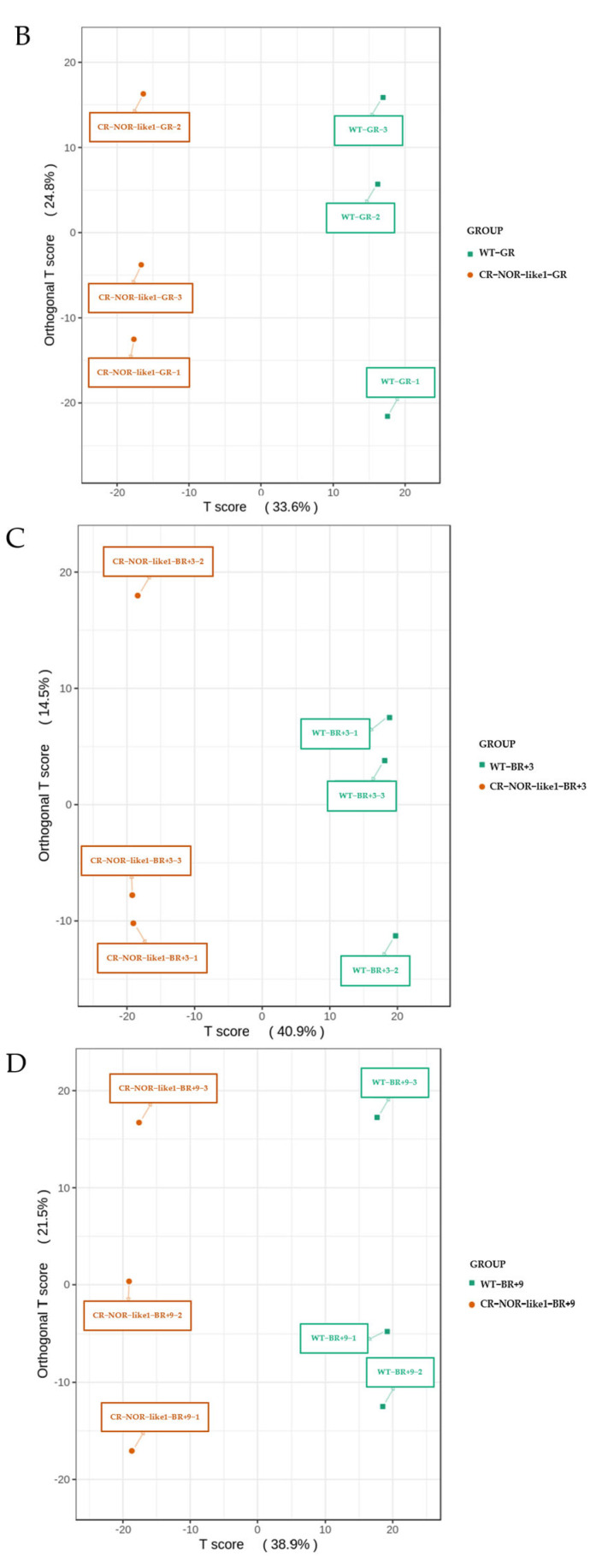
(**A**) PCA results of the overall sample. Each dot indicates a sample, and one color indicates the same group of samples. The closer the distribution of sample points, the more similar the types and levels of metabolites in the samples. Mix is the quality-control sample. PC1, principal component 1; PC2, principal component 2. Explained variants PC1: 27.41%, PC2: 16.04%. (**B**) OPLS-DA model-score scatterplot in GR; (**C**) OPLS-DA model-score scatterplot in BR+3; (**D**) OPLS-DA model-score scatterplot in BR+9, T score indicates the predicted principal component, and the horizontal coordinate direction shows the gap between groups. Orthogonal T score indicates the orthogonal principal component, and the vertical coordinate direction shows the gap within groups. Percentage indicates the degree of explanation of this component to the dataset.

PCA analysis was performed to investigate the trend of separation between the groups and the existence of differences between the samples within the groups. The PCA results (Figure 1A) show that the quality-control samples were well aggregated, demonstrating the good stability of the experimental method. Moreover, the sample points in each group were relatively well concentrated, suggesting good sample reproducibility at each developmental-period point for both tomatoes, and the distances between all groups were relatively dispersed, which indicates that the *NOR-like1* gene editing produced a more significant differential change in the metabolites. The scatter plot of the OPLS-DA model scores (Figure 1B–D) reveals significant differences between each of the two sample groups, and the samples were all within the confidence interval, indicating that there are significantly different metabolites between wild-type and CR-NOR-like1 tomatoes in the same developmental stage, which can be used for subsequent differential-component analyses.

### 3.2. Differential-Metabolite (DEM) Identification

**Figure 2:**
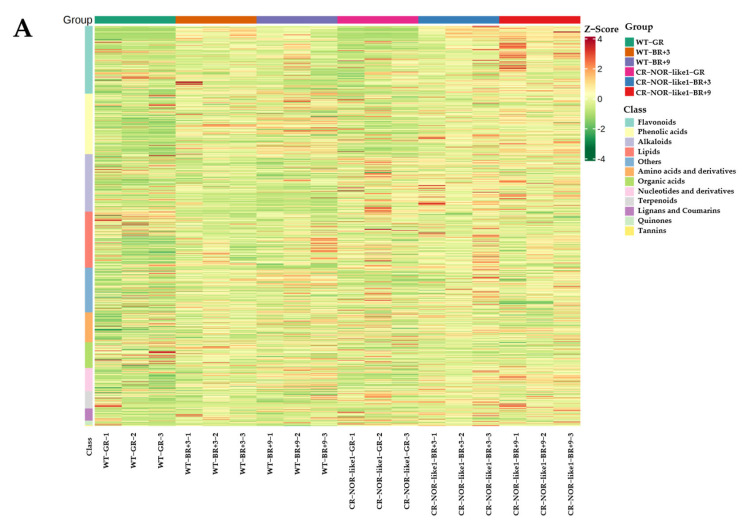

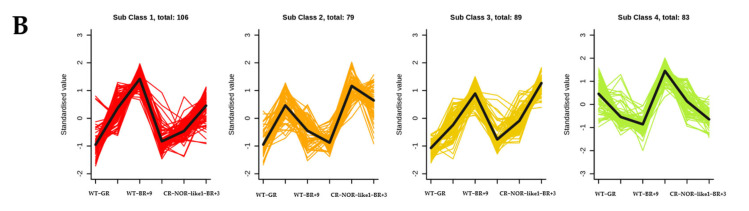
(**A**) Cluster heat map of differential-metabolite content; horizontal is the sample name, vertical is the metabolite information, Group is the grouping, Class is the substance classification, and different colors are the values obtained after normalization of the relative content (red represents high content, green represents low content). (**B**) K-means clustering diagram of differential metabolites; the horizontal coordinate indicates the sample name, the vertical coordinate indicates the normalized relative metabolite content, Sub Class represents the metabolite class number with the same trend of change, and total represents the number of metabolites in the class as.

**Figure 3:**
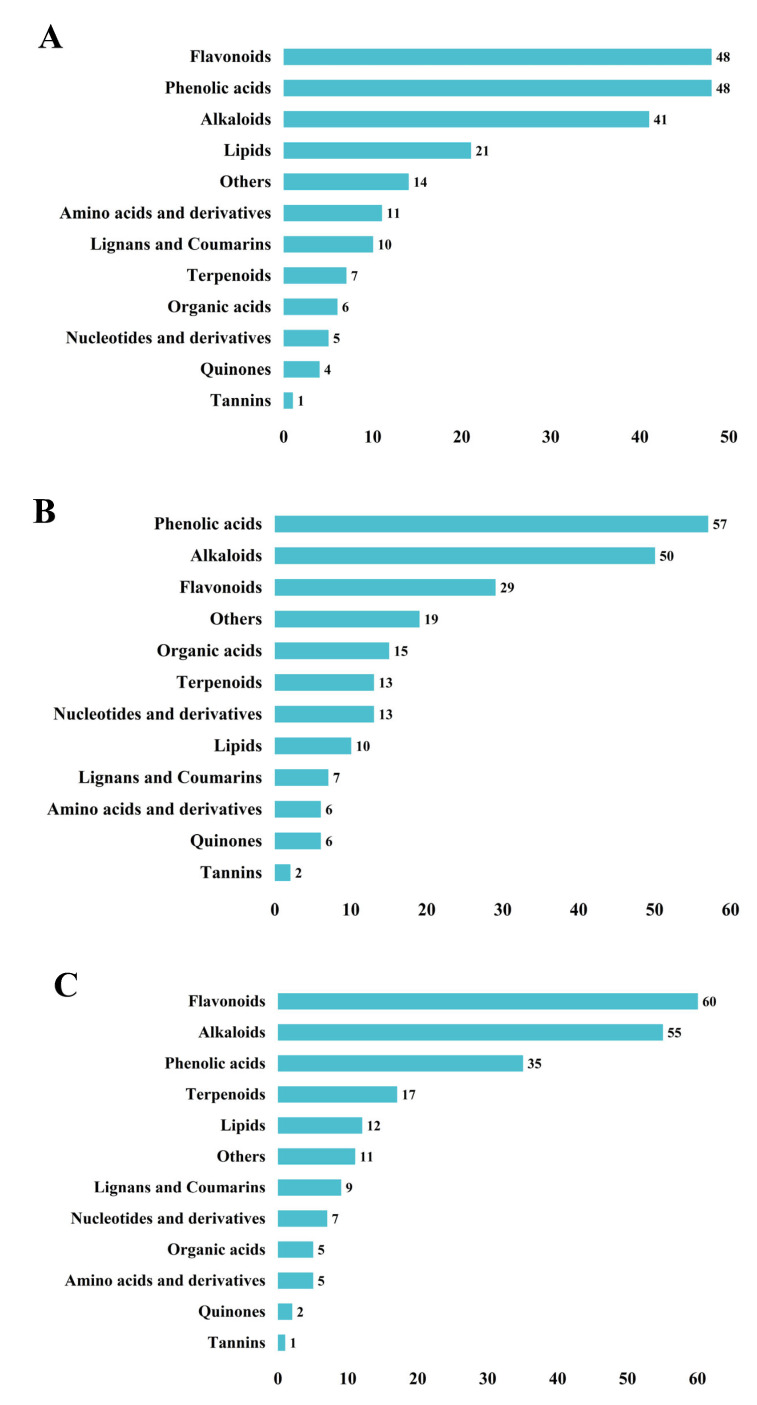
Identified metabolites in the (**A**) GR, (**B**) BR+3, and (**C**) BR+9.

To investigate the differential effects of the *NOR-like1* gene on metabolites in the ripening stage of tomato, three groups of samples were analyzed for significantly different metabolites in three developmental periods before and after *NOR-like1* gene-editing treatment, using FC > 2 or FC < 0.5, *p* < 0.05, and VIP > 1 as selection criteria. Firstly, cluster heat-map analyses (Figure 2A) and k-means cluster analyses (Figure 2B) were applied to the DEMs, and 620 DEMs were grouped in eight clusters. A total of 216 DEMs were detected for WT-GR vs. CR-NOR-like1-GR (Figure 3A), including 177 upregulated and 39 downregulated, containing 48 flavonoids (41 up- and 7 downregulated), 48 phenolic acids (40 up- and 8 downregulated), 41 alkaloids (40 up- and 1 downregulated), 21 lipids, 11 amino acids and their metabolites, 10 lignans and coumarins, 6 organic acids, 7 terpenoids, 5 nucleotides and their derivatives, 4 quinones, and 1 tannin. Altogether, 227 DEMs were measured for WT-BR+3 vs. CR*-*NOR-like1-*B*R+3 (Figure 3B), of which 144 were upregulated and 83 were downregulated, containing 57 phenolic acids (25 up- and 32 downregulated), 50 alkaloids (46 up- and 4 downregulated), 29 flavonoids (18 up- and 11 downregulated), 15 organic acids, 13 terpenoids, 13 nucleotides and their derivatives, 10 lipids, 7 lignans and coumarins, 6 amino acids and their metabolites, 6 quinones, and 2 tannins. A series of 219 DEMs was measured for WT-BR+9 vs. CR-NOR-like1-BR+9 (Figure 3C), of which 179 were upregulated and 40 were downregulated, containing 60 flavonoids (58 up- and 2 downregulated), 55 alkaloids (53 up- and 2 downregulated), 35 phenolic acids (19 up- and 16 downregulated), 17 terpenoids, 12 lipids, 9 lignans and coumarins, 7 nucleotides and their derivatives, 5 organic acids, 5 amino acids and their metabolites, 2 quinones, and 1 tannin. It can be seen that the changes in alkaloids, phenolic acids, and flavonoids were significantly and predominantly upregulated at all three stages; thus, it is suggested that NOR-like1 has a notable influence on these three metabolites during the ripening period of tomato.

**Figure 4:**
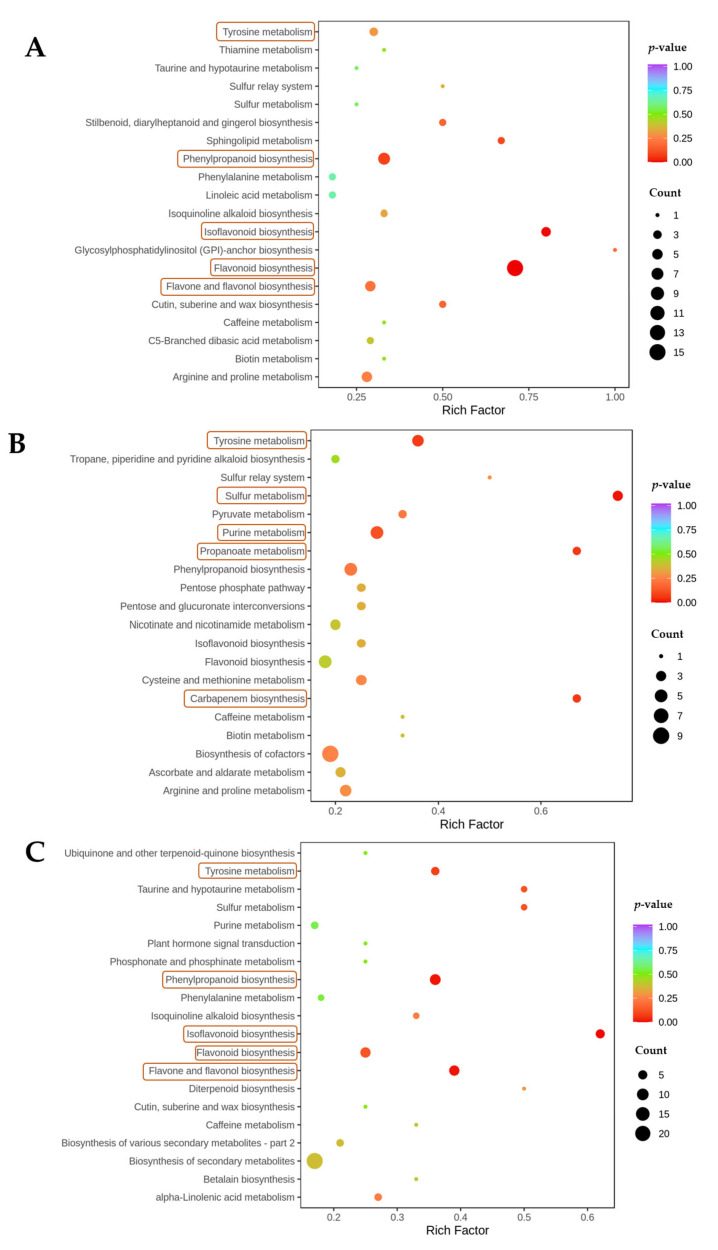
Statistics of metabolomic KEGG enrichment in (**A**) GR, (**B**) BR+3, and (**C**) BR+9. The horizontal coordinate indicates the rich factor of each pathway, the vertical coordinate is the name of the pathway, the color of the dot reflects the *p*-value size, and the redder the color, the more significant the enrichment. The size of the dots represents the number of differential metabolites enriched. Enrichment is significant in the pathway labeled yellow.

The metabolome was analyzed for KEGG-pathway enrichment, and the top 20 pathways with the most significant enrichment were also analyzed by forming differential-enrichment bubble plots (Figure 4). The number of metabolites annotated by KEGG during the GR was 326, mainly distributed in 49 metabolic pathways and significantly enriched in flavonoid biosynthesis, isoflavone biosynthesis, phenylpropanoid biosynthesis, flavonoid and flavonol biosynthesis, tyrosine metabolism, etc. The number of metabolites annotated by KEGG during the BR+3 was 340, mainly distributed in 53 metabolic pathways and significantly enriched in sulfur metabolism, tyrosine metabolism, purine metabolism, propionate metabolism, carbapenem metabolism, etc. The number of KEGG annotated metabolites in the BR+9 was 340, distributed mainly in 36 metabolic pathways, with significant enrichment in isoflavone biosynthesis, flavonoid and flavonol biosynthesis, flavonoid biosynthesis, phenylpropanoid biosynthesis, purine metabolism, etc. It was observed that the flavonoid and phenylpropanoid pathways were significantly enriched particularly in GR and BR+9.

### 3.3. An Overview of RNA-Seq Data

High-quality libraries reflecting transcripts expressed in three developmental stages of wild-type and CR-NOR-like1 tomato (six strains, each with three biological replicates, with a total of 18 samples) were analyzed by RNA-seq on the Illumina HiSeq platform. Clean reads for follow-up analysis were derived by filtering the raw data and checking the sequencing rate of error, as well as the GC content profile (Table S1). A, B, and C represent GR, BR+3, and BR+9 of wild-type tomatoes, respectively; D, E, and F represent GR, BR+3, and BR+9 of CR-NOR-like1 tomatoes, respectively.

### 3.4. Differentially Expressed Gene (DEG) Identification

**Figure 5:**
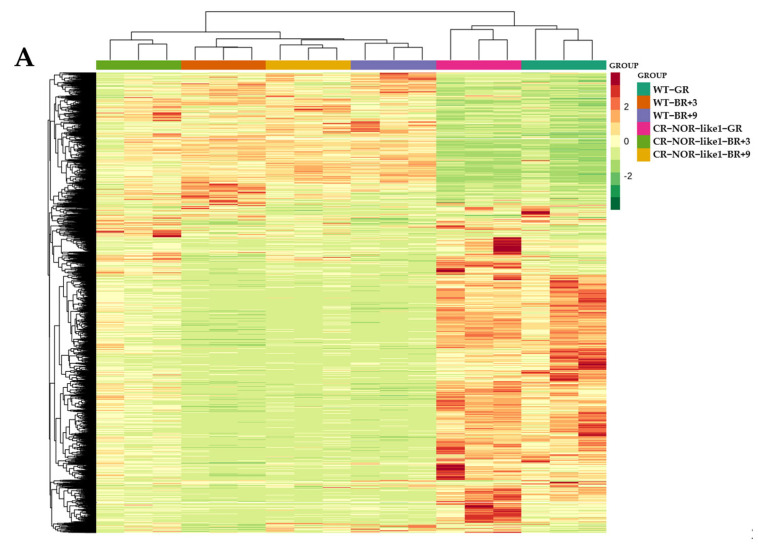

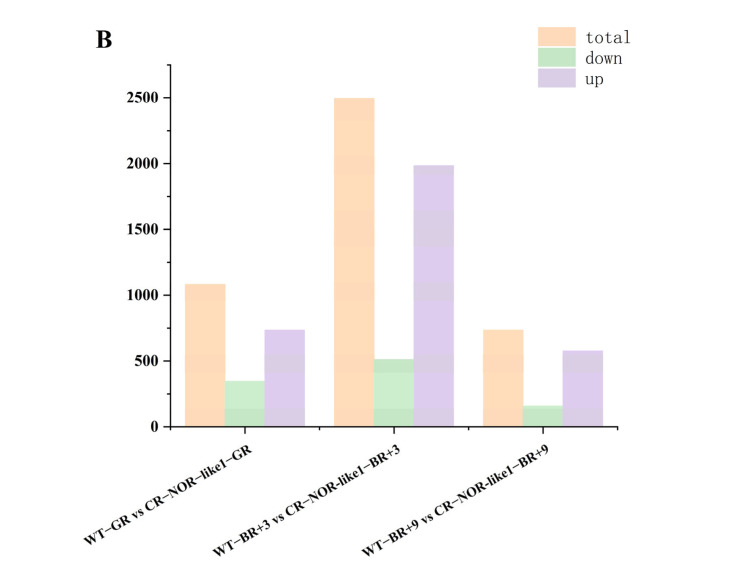
(**A**) The differentially expressed genes in the hierarchical clustering heat map; horizontal coordinates indicate sample names and hierarchical clustering results, and vertical coordinates indicate differential genes and hierarchical clustering results. Red indicates high expression; green indicates low expression. (**B**) Numbers of differentially expressed genes.

**Figure 6:**
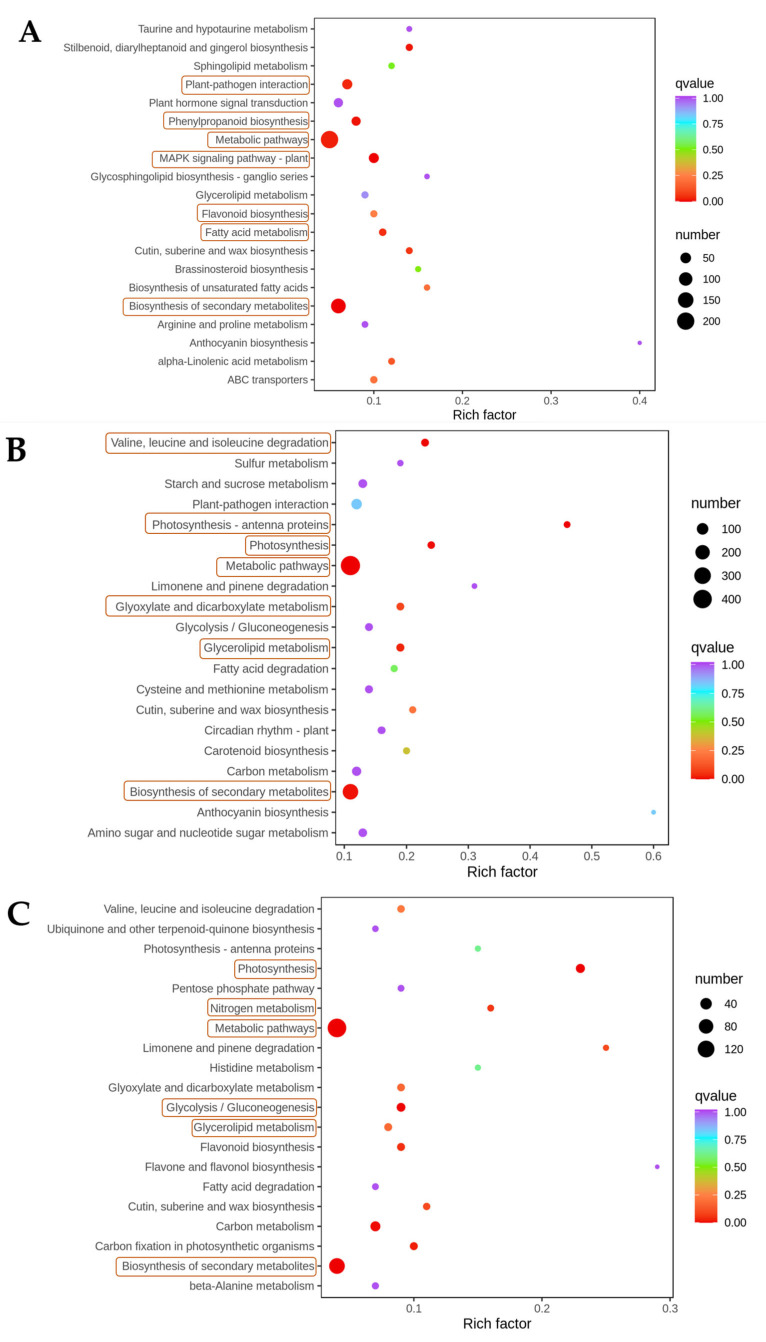
Statistics of transcriptomic KEGG enrichment in the (**A**) GR, (**B**) BR+3, and (**C**) BR+9. The vertical coordinate represents the KEGG pathway. The horizontal coordinate indicates the rich factor; the larger the rich factor, the greater the enrichment. The larger the dot, the greater the number of differential genes enriched in the pathway. The redder the color of the dot, the more significant the enrichment. Enrichment is significant in the pathway labeled yellow.

To identify DEGs in different tomato-ripening processes (the reference genome was from the NCBI database), we first investigated gene-expression patterns under different treatment conditions, centered and normalized the FPKM of differential genes, and then extracted the centralized and normalized FPKM values of the differential genes and analyzed them by hierarchical clustering (Figure 5A), which showed differential expression of a multitude of genes among samples. Furthermore, to find DEGs between samples and to analyze them for other functions, |log_2_Fold Change| ≥ 1 and FDR < 0.05 were taken as conditions for screening DEGs (Figure 5B). In A vs. D, 736 genes were upregulated and 346 genes were downregulated; in B vs. E, 1984 genes were upregulated and 511 genes were downregulated; and in C vs. F, 577 genes were upregulated and 158 genes were downregulated. To further identify the metabolic pathways participating in DEGs, we performed a KEGG-pathway enrichment analysis (Figure 6), and metabolic pathways and signal transduction pathways were identified in which DEGs were significantly enriched.

The DEGs in WT-GR vs. CR-NOR-like1-GR mapped to 111 KEGG pathways, enriched primarily in metabolic pathways (201, 54.92%) and secondary metabolite biosynthesis (129, 35.25%). In addition to these two pathways, it was also significantly enriched in plant–pathogen interaction, MAPK signaling pathway–plant, phenylpropanoid biosynthesis, fatty-acid metabolism, and flavonoid biosynthesis. The DEGs in WT-BR+3 vs. CR-NOR-like1-BR+3 mapped to 132 KEGG pathways. The representative pathways were also metabolic pathways (445, 53.36%) and biosynthesis of secondary metabolites (247, 29.62%); the rest of the significantly enriched pathways were photosynthesis, photosynthesis-antenna proteins, valine, leucine and isoleucine degradation, glycerolipid metabolism, and glyoxylate and dicarboxylate metabolism. The DEGs in WT-BR+9 vs. CR-NOR-like1-BR+9 mapped to 117 KEGG pathways and were similarly enriched mainly in the biosynthesis of secondary metabolites (159, 61.87%) and metabolic pathways (100, 38.91%). Other significant enrichment pathways were carbon metabolism, photosynthesis, glycolysis/gluconeogenesis, flavonoid biosynthesis, and nitrogen metabolism. The results illustrate that NOR-like1 significantly influences expression levels at different developmental stages involving metabolism, organic systems, and environmental-information processing, with a particularly pronounced effect on metabolism. Flavonoid biosynthesis and phenylpropanoid biosynthesis were more significantly enriched in GR and BR+9 compared to BR+3.

### 3.5. Effect of NOR-like1 on Alkaloids

A total of 83 distinct alkaloids were identified in the three developmental stages of wild-type and CR-NOR-like1 tomatoes (Table S2), and the content of alkaloids was BR9 > BR3 > GR. In CR-NOR-like1 tomatoes, there were 40 increases and 1 decrease during the GR period, 47 increases and 3 decreases during the BR+3 period, and 53 increases and 2 decreases during the BR+9 period. A total of 19 were significantly different in all three ripening stages. Two of these alkaloids (N-acetylputrescine, agmatine) and three phenolamines (p-coumaroylputrescine, N-feruloylputrescine, N-feruloylagmatine) were annotated to the arginine- and proline-metabolism (ko0330) pathways.

There were 16 DEGs identified in the arginine- and proline-metabolism pathways (Table 1). Compared to wild-type tomatoes, CR-NOR-like1 tomatoes had five upregulated and three downregulated DEGs during GR, six upregulated and two downregulated DEGs during BG+3, and three upregulated and three downregulated DEGs during BR+9. Among them, Ami, ODC, adc1, and P5CS were remarkable changes only in GR; PDH was significantly changed in BR+3 only; and AST was significantly changed in BR+9 alone, whereas 1 ALDH and 1 CPA were significantly different in all three ripening stages.

### 3.6. Effect of NOR-like1 on Phenolic Acids

In total, 85 separate phenolic acids in wild-type tomatoes and CR-NOR-like1 tomatoes at three developmental stages were identified (Table S3), and the relative content of phenolic acid was GR > BR3 > BR9. The CR-NOR-like1 tomatoes had 40 increases and 8 decreases in the GR period, 24 increases and 33 decreases in the BR+3 period, and 19 increases and 16 decreases in the BR+9 period. A total of 17 of these phenolic acids changed significantly at all three ripening stages, and 10 of these were increased in CR-NOR-like1 tomatoes, including 4-aminosalicylic acid, isoferulic acid, ferulic acid, methyl caffeate, p-hydroxycinnamic acid p-hydroxyphenethylamine, gallic acid-4-o-glucoside,1-o-feruloylquinic acid, 5-o-feruloylquinic acid, benzyl-(2″-o-xylosyl) glucoside, and osmanthuside H [2-(4-hydroxyphenyl) ethyl-β-D-apiosyl-(1 → 6)-β-D-glucoside]. Of these, 18 showed significant variation only in the GR, 25 only in the BR+3, 4 only in the BR+9, and 18 in all three periods. The major differential metabolic pathway involved in phenolic acids was phenylpropanoid biosynthesis (ko0940), with 12 phenolic acids annotated in this pathway. All seven of the DEMs involved in the GR were significantly upregulated. BR+3 had 5 DEMs, with two upregulated and three downregulated. BR+9 contained eight DEMs, including five upregulated and three downregulated.

There were 60 related DEGs characterized in the phenylpropanoid biosynthesis pathway (Table 2). Compared to wild-type tomatoes, CR-NOR-like1 tomatoes had 19 upregulated and 11 downregulated during GR, 28 upregulated and 5 downregulated during BR+3, and 9 up-regulated and 3 downregulated during BR. The 1CCR and 1HCT were significantly differentially expressed at all three ripening stages, REF1 and COMT were DEGs only at the GR period, and CAGT was differentially expressed in the BR+3 period only.

### 3.7. Effect of NOR-like1 on Flavonoids

In total, 96 different flavonoids were identified as a result of the three developmental stages in wild-type and CR-NOR-like1 tomatoes (Table S4), and the relative content of flavonoids was BR+9 > GR > BR+3; therefore, it could be concluded that BR+9 may be a critical stage for tomato-flavonoid biosynthesis. The CR-NOR-like1 tomatoes had 41 increases and 7 decreases in the GR, 18 increases and 12 decreases in CR-NOR-like1 tomatoes in the BR+3, and 58 increases and 2 decreases in the BR+9. Eight of them were significantly different at all three developmental stages, including glycitin, calycosin-7-O-glucoside, sieboldin, dihydromarein, chrysoeriol-6,8-di-C-glucoside, chrysoeriol-6-C-glucoside-7-oglucoside, and chrysin,6-C-glucosyl-2 hydroxynaringenin. Combined with the KEGG-pathway enrichment analysis, the flavonoid DEMs were distributed mainly in flavonoid biosynthesis (ko0941), isoflavone biosynthesis pathway (ko0943), and flavonoid and flavonol biosynthesis (ko0944).

The three ripening stages identified 30 DEGs of enzymes associated with flavonoid biosynthesis. (Table 3). Compared to wild-type tomatoes, CR-NOR-like1 tomatoes had nine significantly upregulated and two downregulated during GR, nine were significantly upregulated and four downregulated during BR+3, and 11 upregulated and one downregulated during BR+9. Among them, the HIDH and VR genes were only significantly different in the BR+3 period, whereas the F3H and CHS genes were only significantly different in BR+9.

### 3.8. Correlation Network Analysis

To investigate the effect of NOR-like1 on the regulatory network of alkaloid, phenolic acid, and flavonoid biosynthesis in tomatoes, these three differential metabolites were tested for correlation with differentially expressed genes in three developmental periods, screening DEGs and DEMs with high correlation-coefficient values (r > 0.8) for correlation analysis.

The Ami (LOC101257218) in the metabolic pathway related to alkaloid synthesis showed a high negative correlation with p-coumaroylputrescine (r = −0.832) in the GR period.

According to the results of DEGs and DEMs of phenolic-acid-relevant metabolic pathways, a total of 18 genes were found to show a highly significant correlation with six phenolic acids and one lignan (Table 4). In the GR period, six genes were highly correlated with two phenolic acids, sinapinaldehyde and 5-O-p-coumaroylquinic acid were reduced, and the expression of all six DEGs was upregulated, with BGL (gene-LOC101262919) positively correlated with sinapinaldehyde as well as C4H, HCT, and CAD, which differentially and negatively acted on these two differential metabolites. The BR+3 period contained 15 DEGs highly associated with four phenolic acids, with reduced levels of p-coumaraldehyde, coniferin, and sinapyl alcohol as well as increased levels of ferulic acid. P-coumaraldehyde and coniferin were influenced by CCR, HCT, and TOGT1. Among them, ferulic acid and coniferin were positively regulated by two TOGT1s (LOC101258702, LOC101260915, respectively) and sinapyl alcohol was positively regulated by both HCT (LOC101244961) and BGL (LOC101251735). The remaining DEGs all negatively affected these DEMs to varying degrees. Three genes of BR+9 were highly associated with two phenolic acids and one lignan, and the content of 5-O-p-coumaroylquinic acid and p-coumaraldehyde was reduced in both and negatively correlated with POD, HCT, and 4CL.

Based on the findings of DEGs and DEMs of flavonoid-relevant biosynthesis pathways, a total of eight genes showed a high correlation with 15 flavonoids (Table 5). Six structural genes showed a higher correlation with 10 flavonoids and two phenolic acids in the GR period, and all 12 DEMs were upregulated (5 flavanones, 3 chalcones, 2 flavanonols, 1 flavone, 1 phenolic acid)—hesperetin, homoeriodictyol, phloretin, butin, aromadendrin, naringenin chalcone, hesperetin-7-O-glucoside, chrysin, trans-5-O-(p-coumaroyl) shikimate, eriodictyol, luteolin, and phlorizin—and were highly correlated with six DEGs. Among them, C4H (LOC101262919) was positively associated with luteolin, hesperetin, homoeriodictyol, phloretin, butin, aromadendrin, naringenin chalcone, and pinobanksin. The remaining genes—CCOAOMT, CHI, and HCT—all negatively interacted with each of the DEMs in the network to varying degrees. The BR+3 period had one gene showing a higher correlation with three flavonoids, all three DEMs were reduced (3 flavanones), and hesperetin, homoeriodictyo, and isosakuranetin were all positively associated with HCT (LOC101244961). Three genes of BR+9 showed a high correlation with three flavonoids (1 flavones, 1 chalcones, 1 flavanones) and one phenolic acid. HCT (LOC101252161) was negatively correlated with 5-O-p-coumaroylquinic acid, whereas luteolin, isoliquiritigenin, and eriodictyol were positively correlated with CHS (CHS1) and all upregulated. Luteolin was also correlated positively with C4H (LOC101262919).

## 4. Discussion

Within the present study, we integrated widely targeted metabolomic and transcriptomic analyses revealing significant effects of *NOR-like1* gene editing on alkaloids, phenolic acids, and flavonoids in tomato and identified relevant genes engaged in these different metabolites. To understand the effects of *NOR-like1* gene editing, the pathways associated with these substances were screened for DEMs and DEGs based on the findings of KEGG enrichment analysis, and correlations were performed for DEMs and DEGs in pathways with correlations higher than 0.8 (a correlation coefficient higher than 0.8 indicates high correlation).

Steroid alkaloids were the major alkaloid types in the tomatoes, with 27 in total, and all of them were upregulated. The most significantly upregulated alkaloids were γ-solanine (Log_2_FC = 13.07) in BR+3 and β2-tomatine (Log_2_FC = 12.35) in BR+9; these two alkaloids increased much more than the rest, and they all belonged to steroid alkaloids. Research related to Solanaceae has mainly focused on α-tomatine and α-kynurenine, with few reports on γ-solanine and β2-tomatine, both of which aid plants to defend themselves against pathogens and herbivores via their bitterness and toxicity, and are enriched significantly in leaves, roots, and immature green tomatoes [16]. However, *NOR-like1* gene editing produced a very significant positive effect on the late-ripening (BR+3, BR+9) tomatine. A total of five alkaloids were annotated to arginine and proline metabolism. Agmatine is a metabolite associated with arginine and proline metabolism, whereas n-acetylputrescine, p-coumarinylputrescine, ferulic putrescine, and n-ferulic agmatine are related derivatives [32]. Analyses of correlation revealed that only Ami (LOC101257218) was highly negatively associated with p-coumaroylputrescine and that p-coumaroylputrescine was increased in tomatoes (Log_2_FC = 3.20). Ami is an important enzyme in arginine and proline metabolism, and the downregulation of Ami facilitated the accumulation of arginine and proline [33]. Consequently, these alkaloids associated with arginine and proline metabolic pathways were also accumulated. Based on these results, we came to the conclusion that NOR-like1 positively affects alkaloid synthesis in tomatoes, especially late ripening. Therefore, we hypothesize that the changes in arginine and proline metabolic pathways as also part of plant defense mechanisms.

Changes in the gene-expression levels of the phenylpropanoid biosynthesis pathway correlated with variation in lignin, phenolic acids, and flavonoids, whereas changes in the phenylpropanoid biosynthesis in CR-NOR-like1 tomatoes mainly involved alterations in phenolic acids. These substances are initially converted from phenylalanine to cinnamic acid by deamination under the action of PAL, followed by hydroxylation to p-coumaric acid catalyzed by C4H, and eventually converted to p-coumaroyl CoA by the addition of a CoA to p-coumaric acid catalyzed by 4CL, finally entering the phenolic-acid pathway to produce p-coumaroyl quinic acid, caffeic acid, ferulic acid, etc., which exist in the plant in a free state and combined with esters or glycosides [14,34,35,36]. POD, CCR, and BGL were shown to be important enzymes in the plant-defense response [37], and all three enzymes were predominantly upregulated in CR-NOR-like1 tomatoes. Sinapinaldehyde was the most upregulated of all alkaloids in the GR period (Log_2_FC = 9.84) and it was the precursor of sinapyl-alcohol synthesis in all three periods; however, sinapyl alcohol was reduced in BR+3 under the negative regulation of BGL (LOC101251735) (Log_2_FC = −2.35). Because CAD is the enzyme capable of reducing sinapinaldehyde to the corresponding sinapyl alcohol [38], we presumed that the decrease in sinapyl-alcohol content could have been due to significant negative regulation of sinapinaldehyde by CAD (LOC101253340). Ferulic acid is a key metabolite annotated to the phenylpropanoid biosynthesis and is positively regulated at BR+3 by TOGT1 (LOC101260915). A natural production most commonly found in tomatoes, ferulic acid is widespread in the cell wall and has free radical scavenging and antiviral functions [39]. NOR-like1 significantly affects key enzymes in the phenylpropanoid biosynthesis pathway, positively influencing phenolic-acid components.

Among all the flavonoids that were significantly changed, flavonols (28), flavones (18), and flavanones (16) accounted for more than half of all flavonoid differential metabolites. In the GR period, chrysin, naringenin chalcone, luteolin, etc.; chrysin in BR+3; and acacetin, chrysin, wogonin, etc. in BR+9 were where the flavonoids increased most obviously. Of these, chrysin was upregulated extremely significantly in all three periods (Log_2_FC = 12.17, GR; Log_2_FC = 13.81, BR+3; Log_2_FC = 8.07, BR+9). Chrysin has been shown to have antioxidant capacity and can scavenge free radicals, is anti-inflammatory, and has demonstrated other activities [40], but it has been less studied in tomatoes. The genes associated with flavonoid biosynthesis are mainly classified into structural and regulatory genes [41]—for instance, CHS, FLS, F3H, F3′H, C4H, etc. C4H plays an important role in flavonoid biosynthesis [42,43,44]. Correlation analysis showed that C4H at all three ripening stages positively regulated flavonoids including luteolin, hesperetin, homoeriodictyol, phloretin, butin, aromadendrin, naringenin chalcone, and pinobanksin. CHS, FLS, and CHI are key enzymes in flavonoid biosynthesis. The first key speed-limiting enzyme in the flavonoid biosynthetic is CHS [45], whereas CHS and FLS can synergistically upregulate the biosynthesis of flavonols in tomatoes [46]. Both genes were mainly expressed as upregulated in BR+9, whereas the content of flavonols in BR+9 was significantly increased (20 increased and 1 decreased). CHI is the second key speed-limiting enzyme in flavonoid biosynthesis [47]. Naringin chalcone can be isomerized in the presence of CHI to produce naringin, a procedure that may alternatively occur spontaneously in the absence of active CHI [16]. In the present study, CHI was down-regulated in GR, whereas naringenin chalcone and naringenin were very significantly upregulated in GR. Apart from that, F3H, or F3′H, is also a critical enzyme in flavonoid biosynthesis. F3H acts on naringin and eriodictyol, resulting in the substitution of the C3 position with a hydroxyl group. This leads to the formation of the corresponding dihydroflavonols, i.e., dihydrokaempferol (DHK), dihydroquercetin (DHQ), and DHQ, which can be obtained by catalyzing DHK in the presence of F3′H [14]. Naringenin, eriodictyol, and DHK for GR as well as eriodictyol and DHQ for BR+9 were significantly up-regulated, as shown in Table S4. *NOR-like1* gene editing greatly affected the critical enzymes for flavonoid biosynthesis, and the variation in flavonoid metabolites was most obvious from the data.

The present results indicate that NOR-like1 dramatically affected gene-expression levels involved in metabolism, organic systems, and environmental-information processing in the different developmental stages, especially on alkaloids, phenolic acids, and flavonoids, with flavonoids being the most dramatic change. Highly relevant key metabolites and key regulatory genes were further screened by correlation analysis. Ami in the arginine and proline metabolic pathways; PAL, C4H, 4CL, and CAD in the phenylpropane biosynthesis; and CHS, FLS, F3H, F3′H, and C4H in the flavonoid pathway all had significant regulatory effects on the accumulation of alkaloids, phenolic acids, and flavonoid metabolites. It was demonstrated that under the same inherited background it is possible to store fruits with higher overall antioxidant capacity longer than those of lower antioxidant capacity, that tomato fruits with higher antioxidant ability show slower overripening [48], and that phenolics and alkaloids also have a significant effect on biotic–biotic resistance. Accordingly, we hypothesized that *NOR-like1* gene editing would enhance antioxidant capacity and cause delayed ripening by upregulating alkaloid, phenolic acid, and flavonoid accumulation during tomato ripening. The present study lays the foundation for extending fruit longevity and shelf life as well as cultivating stress-resistant plants, and also provides directions for further studies on the mechanisms of NOR-like1 transcription-factor effects on metabolites in tomatoes. Furthermore, it enriched the study of NAC gene function and regulation in tomatoes and initially revealed the effect of *NOR-like1* gene editing on the accumulation of alkaloids, phenolic acids, and flavonoid metabolites in tomato. The effect of NOR-like1 on the metabolism of alkaloids, phenolic acids, and flavonoids during tomato ripening needs to be further investigated—for instance, with antioxidant assays or combined with proteomic approaches—to enrich our studies and explore more deeply the regulatory mechanism of NOR-like1 transcription factor.

## Figures and Tables

**Table 1:** List of DEGs related to alkaloids.

Gene Name	Gene ID	WT-GR vs. CR-NOR-like1-GR			WT-BR+3 vs. CR-NOR-like1-BR+3			WT-BR+9 vs. CR-NOR-like1-BR+9		
		Log_2_FC	*p*-Value	Type	Log_2_FC	*p*-Value	Type	Log_2_FC	*p*-Value	Type
Aldehyde dehydrogenase (ALDH)	LOC104645747	−1.03	3.59 × 10^−3^	Down	2.50	9.62 × 10^−13^	Up	2.58	1.38 × 10^−4^	Up
	SlADH2B7d	−1.64	2.12 × 10^−26^	Down	--	--	--	--	--	--
	LOC101250474	--	--	--	−2.10	8.19 × 10^−10^	Down	−1.77	5.66 × 10^−6^	Down
	SlADH2B7d	--	--	--	2.35	5.23 × 10^−13^	Up	--	--	--
	SlADH3F1a	--	--	--	−1.57	1.62 × 10^−13^	Down	--	--	--
	SlALDH2B7a	--	--	--	3.03	4.59 × 10^−14^	Up	1.45	5.86 × 10^−6^	Up
	SlALD3H1	--	--	--	--	--	--	−1.11	4.09 × 10^−7^	Down
Amidase (Ami)	LOC101257218	−1.57	5.71 × 10^−4^	Down	--	--	--	--	--	--
	LOC101260379	1.97	1.72 × 10^−3^	Up	--	--	--	--	--	--
Arginase (ARG2)	ARG2	2.27	4.39 × 10^−4^	Up	3.75	1.25 × 10^−3^	Up	--	--	--
Ornithine decarboxylase (ODC)	ODC	1.90	1.19 × 10^−3^	Up	--	--	--	--	--	--
Arginine decarboxylase (adc1)	adc1	1.98	5.37 × 10^−6^	Up	--	--	--	--	--	--
N-carbamoylputrescine amidase (CPA)	LOC101268110	9.02	1.53 × 10^−12^	Up	9.39	4.97 × 10^−14^	Up	7.65	1.61 × 10^−9^	Up
Delta-1-pyrroline-5-carboxylate synthetase (P5CS)	LOC101244293	−1.16	2.50 × 10^−10^	Down	--	--	--	--	--	--
Proline dehydrogenase (PDH)	PDH	--	--	--	2.33	8.58 × 10^−15^	Up	--	--	--
Aspartate aminotransferase (AST)	LOC101244012	--	--	--	--	--	--	−1.15	4.13 × 10^−6^	Down

**Table 2:** List of DEGs related to phenolic acids.

Gene Name	Gene ID	WT-GR vs. CR-NOR-like1-GR			WT-BR+3 vs. CR-NOR-like1-BR+3			WT-BR+9 vs. CR-NOR-like1-BR+9		
		Log_2_FC	*p*-Value	Type	Log_2_FC	*p*-Value	Type	Log_2_FC	*p*-Value	Type
Phenylalanine ammonia-lyase (PAL)	PAL5	2.71	2.75 × 10^−4^	Up	--	--	--	2.52	1.50 × 10^−6^	Up
	LOC101243631	--	--	--	--	--	--	1.18	1.62 × 10^−4^	Up
	PAL3	--	--	--	--	--	--	1.27	2.20 × 10^−6^	Up
Trans-cinnamate 4-monooxygenase (C4H)	LOC101262919	3.28	2.83 × 10^−6^	Up	--	--	--	−2.45	1.56 × 10^−6^	Down
	LOC101244496	--	--	--	--	--	--	1.15	1.52 × 10^−3^	Up
4-coumarate—CoA ligase (4CL)	LOC101251197	−4.24	1.46 × 10^−4^	Down	--	--	--	--	--	--
	LOC101251363	--	--	--	1.06	1.75 × 10^−3^	Up	1.71	1.65 × 10^−3^	Up
Cinnamoyl-CoA reductase (CCR)	CCR2	−1.86	1.39 × 10^−6^	Down	--	--	--	--	--	--
	LOC101246651	−1.59	1.55 × 10^−5^	Down	1.47	6.13 × 10^−5^	Up	2.11	4.65 × 10^−5^	Up
	LOC101264879	−2.04	1.23 × 10^−4^	Down	--	--	--	1.71	2.82 × 10^−6^	Up
	LOC101250958	--	--	--	1.16	3.84 × 10^−3^	Up	--	--	--
	LOC101262601	--	--	--	2.71	8.51 × 10^−14^	Up	--	--	--
	LOC101265652	--	--	--	1.32	9.78 × 10^−5^	Up	--	--	--
	PAR2	--	--	--	1.17	2.15 × 10^−3^	Up	--	--	--
Cinnamyl-alcohol dehydrogenase (CAD)	LOC112940682	6.67	7.36 × 10^−5^	Up	--	--	--	--	--	--
	LOC101253340	−3.12	1.84 × 10^−3^	Down	2.51	4.30 × 10^−3^	Up	--	--	--
	LOC101250635	--	--	--	1.45	4.30 × 10^−4^	Up	--	--	--
	LOC101265606	--	--	--	--	--	--	−2.27	3.28 × 10^−3^	Down
Peroxidase (POD)	LOC101244376	−7.59	8.55 × 10^−7^	Down	--	--	--	--	--	--
	LOC101251503	5.18	4.85 × 10^−13^	Up	--	--	--	--	--	--
	LOC101253377	1.34	2.53 × 10^−3^	Up	--	--	--	--	--	--
	LOC101257228	4.96	5.63 × 10^−11^	Up	--	--	--	--	--	--
	LOC101263035	−1.25	3.36 × 10^−3^	Down	--	--	--	--	--	--
	LOC101258529	--	--	--	2.72	1.18 × 10^−3^	Up	--	--	--
	LOC101267754	--	--	--	2.18	3.45 × 10^−3^	Up	--	--	--
	LOC101268153	--	--	--	2.58	4.52 × 10^−4^	Up	--	--	--
	TAP2	--	--	--	1.82	2.65 × 10^−3^	Up	--	--	--
	TMP1	--	--	--	2.76	4.28 × 10^−18^	Up	--	--	--
	LOC101253648	--	--	--	--	--	--	−1.45	6.78 × 10^−5^	Down
Caffeoyl-CoA O-methyltransferase (CCoAOMT)	LOC101253032	2.12	8.52 × 10^−10^	Up	--	--	--	--	--	--
	LOC101252203	--	--	--	2.70	1.47 × 10^−6^	Up	--	--	--
Beta-glucosidase (BGL)	LOC101249847	3.83	4.29 × 10^−7^	Up	--	--	--	--	--	--
	LOC101246223	−2.81	2.96 × 10^−4^	Down	--	--	--	--	--	--
	LOC101251735	--	--	--	−1.51	2.27 × 10^−5^	Down	--	--	--
	LOC101256510	--	--	--	1.66	3.21 × 10^−4^	Up	--	--	--
	LOC101256717	--	--	--	1.37	1.00 × 10^−4^	Up	--	--	--
	LOC101265077	--	--	--	1.24	2.71 × 10^−8^	Up	--	--	--
	LOC101260057	--	--	--	2.59	1.60 × 10^−6^	Up	--	--	--
	LOC101266643	--	--	--	2.57	1.65 × 10^−6^	Up	--	--	--
Feruloyl-CoA ortho-hydroxylase (FC2′H)	LOC101252918	2.75	8.03 × 10^−6^	Up	2.05	1.39 × 10^−3^	Up	--	--	--
Coumaroylquinate(coumaroylshikimate) 3′-monooxygenase (C3′H)	LOC101246092	1.77	3.55 × 10^−3^	Up	1.17	3.53 × 10^−3^	Up	--	--	--
Coniferyl-aldehyde dehydrogenase (REF1)	LOC101247788	1.27	1.63 × 10^−3^	Up	--	--	--	--	--	--
Shikimate O-hydroxycinnamoyltransferase (HCT)	LOC101245886	−2.20	3.09 × 10^−6^	Down	2.30	3.42 × 10^−10^	Up	2.18	3.96 × 10^−5^	Up
	LOC101247305	7.39	1.06 × 10^−4^	Up	--	--	--	--	--	--
	LOC101248087	5.05	3.14 × 10^−8^	Up	--	--	--	--	--	--
	LOC101252161	−2.31	2.14 × 10^−3^	Down	--	--	--	2.28	7.77 × 10^−4^	Up
	LOC101253556	5.90	5.02 × 10^−12^	Up	--	--	--	--	--	--
	LOC101256271	1.60	1.93 × 10^−3^	Up	--	--	--	--	--	--
	LOC101260610	5.16	1.78 × 10^−4^	Up	--	--	--	--	--	--
	LOC101244961	--	--	--	−1.58	1.04 × 10^−3^	Down	--	--	--
	LOC101246106	--	--	--	−1.24	3.17 × 10^−3^	Down	--	--	--
	LOC101266953	--	--	--	1.62	5.32 × 10^−3^	Up	--	--	--
Caffeic acid 3-O-methyltransferase (COMT)	LOC101251452	1.49	5.89 × 10^−4^	Up	--	--	--	--	--	--
Scopoletin glucosyltransferase (TOGT1)	LOC101253350	3.07	6.84 × 10^−8^	Up	--	--	--	--	--	--
	LOC101259704	−1.92	1.37 × 10^−16^	Down	--	--	--	--	--	--
	twi1	1.31	2.54 × 10^−3^	Up	--	--	--	--	--	--
	GAME1	--	--	--	2.32	1.34 × 10^−2^	Up	--	--	--
	LOC101258702	--	--	--	−1.36	3.72 × 10^−4^	Down	--	--	--
	LOC101260915	--	--	--	5.63	1.64 × 10^−10^	Up	--	--	--
Coniferyl-alcohol glucosyltransferase (CAGT)	LOC101256157	--	--	--	−3.10	2.92 × 10^−17^	Down	--	--	--

**Table 3:** List of DEGs related to flavonoids.

Gene Name	Gene ID	WT-GR vs. CR-NOR-like1-GR			WT-BR3 vs. CR-NOR-like1-BR+3			WT-BR9 vs. CR-NOR-like1-BR+9		
		Log_2_FC	*p*-Value	Type	Log_2_FC	*p*-Value	Type	Log_2_FC	*p*-Value	Type
Shikimate O-hydroxycinnamoyltransferase (HCT)	LOC101253556	5.90	5.02 × 10^−12^	Up	--	--	--	--	--	--
	LOC101248087	5.05	3.14 × 10^−8^	Up	--	--	--	--	--	--
	LOC101245886	−2.20	3.09 × 10^−6^	Down	2.30	3.42 × 10^−10^	Up	2.18	3.96 × 10^−5^	Up
	LOC101247305	7.39	1.06 × 10^−4^	Up	--	--	--	--	--	--
	LOC101260610	5.16	1.78 × 10^−4^	Up	--	--	--	--	--	--
	LOC101256271	1.60	1.93 × 10^−3^	Up	--	--	--	--	--	--
	LOC101252161	−2.31	2.14 × 10^−3^	Down	--	--	--	2.28	7.77 × 10^−4^	Up
	LOC101244961	--	--	--	−1.58	1.04 × 10^−3^	Down	--	--	--
	LOC101246106	--	--	--	−1.24	3.17 × 10^−3^	Down	--	--	--
	LOC101266953	--	--	--	1.62	5.32 × 10^−3^	Up	--	--	--
Caffeoyl-CoA O-methyltransferase (CCoAOMT)	LOC101253032	2.12	8.52 × 10^−10^	Up	--	--	--	--	--	--
	LOC101252203	--	--	--	2.70	1.47 × 10^−6^	Up	--	--	--
Trans-cinnamate 4-monooxygenase (C4H)	LOC101262919	3.28	2.83 × 10^−6^	Up	--	--	--	−2.45	1.56 × 10^−6^	Down
	LOC101244496	--	--	--	--	--	--	1.15	1.52 × 10^−3^	Up
Flavonol synthase (FLS)	LOC101260801	3.70	7.56 × 10^−4^	Up	−1.51	4.18 × 10^−3^	Down	--	--	--
	LOC101260380	--	--	--	--	--	--	9.94	2.70 × 10^−16^	Up
	LOC101249699	--	--	--	--	--	--	1.73	2.63 × 10^−5^	Up
Chalcone isomerase (CHI)	CHI1	−1.14	1.42 × 10^−3^	Down	--	--	--	--	--	--
	LOC101266223	--	--	--	--	--	--	2.84	3.54 × 10^−9^	Up
Coumaroylquinate(coumaroylshikimate) 3′-monooxygenase (C3H)	LOC101246092	1.77	3.55 × 10^−3^	Up	1.17	3.53 × 10^−3^	Up	--	--	--
Leucoanthocyanidin dioxygenase (LDOX)	LOC101248628	--	--	--	−3.34	1.70 × 10^−6^	Down	--	--	--
Naringenin 3-dioxygenase (F3H)	F3H	--	--	--	--	--	--	2.51	6.12 × 10^−10^	Up
Flavonoid 3′-monooxygenase (F3′H)	LOC101266618	--	--	--	--	--	--	2.31	2.27 × 10^−9^	Up
Chalcone synthase (CHS)	CHS1	--	--	--	--	--	--	3.13	5.24 × 10^−5^	Up
	CHS2	--	--	--	--	--	--	1.19	8.59 × 10^−4^	Up
2-hydroxyisoflavanone dehydratase (HIDH)	ASH1	--	--	--	2.65	9.81 × 10^−8^	Up	--	--	--
Vestitone reductase (VR)	LOC101264524	--	--	--	4.15	2.20 × 10^−4^	Up	--	--	--
Isoflavone/4′-methoxyisoflavone 2’-hydroxylase(I2′H)	LOC101250559	--	--	--	1.68	9.19 × 10^−3^	Up	1.81	2.91 × 10^−3^	Up
Flavonol-3-O-glucoside/galactoside glucosyltransferase (FG3)	LOC104649610	--	--	--	1.73	1.05 × 10^−5^	Up	--	--	--
	LOC101244316	--	--	--	1.25	1.07 × 10^−3^	Up	4.20	5.14 × 10^−6^	Up

**Table 4:** Result of phenolic-acid correlation network analysis.

Gene Name	Gene ID	Compounds	PCC
HCT	LOC101252161	Sinapinaldehyde	−0.85
		5-O-p-Coumaroylquinic acid	−0.81
	LOC101244961	p-Coumaraldehyde	0.945
BGL	LOC101246223	Sinapinaldehyde	−0.82
		5-O-p-Coumaroylquinic acid	−0.83
	LOC101251735	Sinapyl alcohol	0.837
		p-Coumaraldehyde	0.879
	LOC101265077	p-Coumaraldehyde	−0.89
		Coniferin	−0.84
	LOC101256717	p-Coumaraldehyde	−0.92
		Coniferin	−0.82
C4H	LOC101262919	Sinapinaldehyde	0.881
TOGT1	LOC101259704	Sinapinaldehyde	−0.86
		5-O-p-Coumaroylquinic acid	−0.82
	GAME1	Coniferin	−0.89
		p-Coumaraldehyde	−0.87
	LOC101258702	Coniferin	0.916
		p-Coumaraldehyde	0.897
	LOC101260915	Ferulic acid *	0.803
CAD	LOC101253340	5-O-p-Coumaroylquinic acid	−0.85
	LOC101250635	Coniferin	−0.81
		p-Coumaraldehyde	−0.88
POD	LOC101244376	Sinapinaldehyde	−0.97
	TMP1	p-Coumaraldehyde	−0.94
		Coniferin	−0.93
	TAP2	Coniferin	−0.86
		p-Coumaraldehyde	−0.9
	LOC101253648	7-Hydroxycoumarin	−0.86
		p-Coumaraldehyde	−0.84
CCR	LOC101250958	Coniferin	−0.9
		p-Coumaraldehyde	−0.9
	LOC101265652	p-Coumaraldehyde	−0.85
	PAR2	Coniferin	−0.81
		p-Coumaraldehyde	−0.84
4CL	LOC101251363	Coniferin	−0.82
		5-O-p-Coumaroylquinic acid	−0.84

NOTE: * indicates the presence of an isomer of the substance in the test result.

**Table 5:** Result of flavonoid correlation network analysis.

Gene Name	Gene ID	Compounds	PCC
HCT	LOC101252161	Naringenin chalcone	−0.816
		Pinobanksin	−0.803
		Hesperetin	−0.862
		5-O-p-Coumaroylquinic acid	−0.813
		Phloretin	−0.815
		Butin	−0.821
		Aromadendrin (dihydrokaempferol)	−0.808
		Homoeriodictyol	−0.859
	LOC101253556	Phloretin-2′-O-glucoside (phlorizin)	−0.868
	LOC101247305	Phloretin-2′-O-glucoside (phlorizin)	−0.902
	LOC101244961	Homoeriodictyol	0.832
		Hesperetin	0.818
		Isosakuranetin (5,7-dihydroxy-4′-methoxyflavanone)	0.925
CHI	CHI1	Eriodictyol (5,7,3′,4′-tetrahydroxyflavanone)	−0.846
		Homoeriodictyol	−0.834
		Pinobanksin	−0.823
		Naringenin (5,7,4′-trihydroxyflavanone)	−0.832
		Hesperetin	−0.825
		Aromadendrin (dihydrokaempferol)	−0.82
C4H	LOC101262919	Luteolin (5,7,3′,4′-tetrahydroxyflavone)	0.832
		Naringenin (5,7,4′-trihydroxyflavanone)	0.807
		Naringenin chalcone	0.849
		Phloretin	0.854
		Butin	0.85
		Hesperetin	0.839
		Aromadendrin (dihydrokaempferol)	0.841
		Homoeriodictyol	0.84
		Pinobanksin	0.84
CCOMT	LOC101253032	Phloretin-2′-O-glucoside (Pplorizin)	−0.815
CHS	CHS1	Eriodictyol (5,7,3′,4′-tetrahydroxyflavanone)	0.865
		Isoliquiritigenin	0.832
		Luteolin (5,7,3′,4′-tetrahydroxyflavone)	0.848

## Data Availability

The data supporting the results of this study are included in the present article.

## Supplementary Materials

The following supporting information can be downloaded at: https://www.mdpi.com/article/10.3390/metabo12121296/s1, Table S1: An overview of the RNA-Seq data; Table S2: Differential metabolites of alkaloids; Table S3: Differential metabolites of phenolic acids; Table S4: Differential metabolites of flavonoids.
