# Supplementary material for: Integrated Analysis of Widely Targeted Metabolomics and Transcriptomics Reveals the Effects of Transcription Factor NOR-like1 on Alkaloids, Phenolic Acids, and Flavonoids in Tomato at Different Ripening Stages
Source: Metabolites. 2022 Dec 19;12(12):1296. doi: 10.3390/metabo12121296 (PMC9853326; doi:10.3390/metabo12121296)
Supplement: Supplementary file 1 [file metabolites-12-01296-s001.zip › metabolites-2035548-supplementary.pdf]

Table S1 An overview of the RNA-Seq data

Sample	Raw Reads	Clean Reads	Clean Base(G)	Error Rate (%)	Q20(%)	Q30(%)	GC Content (%)
A1	47639960	43958024	6.59	0.02	98.19	94.29	42.61
A2	43721172	39920288	5.99	0.02	98.19	94.21	42.71
A3	45202246	42184380	6.33	0.02	98.18	94.26	42.97
B1	46316792	42201960	6.33	0.02	98.27	94.44	41.95
B2	48020222	43832332	6.57	0.02	98.3	94.5	42.09
B3	45241968	41157506	6.17	0.03	98.15	94.15	42
C1	43736374	39937784	5.99	0.03	97.92	93.9	41.68
C2	54547450	49991898	7.5	0.03	97.51	93.11	41.88
C3	45393594	41270852	6.19	0.03	97.97	93.95	41.6
D1	45901324	42290544	6.34	0.02	98.2	94.35	42.39
D2	48620036	44684960	6.7	0.02	98.18	94.23	42.95
D3	47530250	44078740	6.61	0.02	98.15	94.18	42.37
E1	45907556	42156602	6.32	0.02	98.29	94.52	42.28
E2	45567900	42074620	6.31	0.02	98.16	94.25	42.16
E3	48470966	44358868	6.65	0.02	98.22	94.24	41.77
F1	45084556	41341148	6.2	0.02	98.24	94.4	42.16
F2	48351552	47567180	7.14	0.03	98	94.08	41.98
F3	45139248	40727818	6.11	0.03	98.05	94.05	41.97

Table S2 Differential metabolites of alkaloids

Class	Index	Compounds	Q1 (Precursor ion)	Q3 (Fragment ion)	Molecular Weight	WT-GR vs. CR-NOR-like1-GR			WT-BR+3 vs. CR-NOR-like1-BR+3			WT-BR+9 vs. CR-NOR-like1-BR+9		
						p-value	Log_2FC	Type	*p*-Value	Log_2FC	Type	*p*-Value	Log_2FC	Type
**Alkaloids**	pme2693	N-Acetylputrescine	1.31×10^2	1.14×10^2	1.30×10^2	4.33×10^{-2}	1.26	Up	--	--	--	--	--	--
	pmb0501	Agmatine	1.31×10^2	1.14×10^2	1.30×10^2	2.01×10^{-2}	1.13	Up	--	--	--	--	--	--
	pme1002	L-Tyramine	1.38×10^2	1.03×10^2	1.37×10^2	1.04×10^{-1}	1.62	Up	7.30×10^{-4}	1.64	Up	4.75×10^{-2}	1.70	Up
	pma0948	Phenylethanolamine	1.38×10^2	1.21×10^2	1.37×10^2	6.69×10^{-2}	1.68	Up	6.19×10^{-4}	1.62	Up	4.34×10^{-2}	1.67	Up
	mws0704	O-Phosphorylethanolamine	1.40×10^2	79.0	1.41×10^2	1.03×10^{-1}	-1.65	Down	--	--	--	--	--	--
	Lmmp002080	N-(4-Aminobutyl) benzamide	1.93×10^2	1.05×10^2	1.92×10^2	1.61×10^{-1}	2.34	Up	5.66×10^{-2}	1.62	Up	1.22×10^{-2}	1.52	Up
	Hmjp005556	(2E)-3-(4-Hydroxyphenyl)-N-[2-(4-hydroxyphenyl) ethyl]-2-propenamide	2.84×10^2	1.47×10^2	2.83×10^2	2.00×10^{-1}	5.51	Up	--	--	--	--	--	--
	HJN013	N-Feruloyloctopamine	3.28×10^2	1.61×10^2	3.29×10^2	5.62×10^{-3}	2.13	Up	1.42×10^{-1}	2.29	Up	1.36×10^{-1}	2.69	Up
	pme2122	Histamine	1.12×10^2	95.0	1.11×10^2	--	--	--	2.73×10^{-2}	1.40	Up	--	--	--
	MWSmce542	(S)-Indoximod	2.19×10^2	1.32×10^2	2.19×10^2	1.09×10^{-1}	1.14	Up	--	--	--	--	--	--
**Plumerane**	pmb0323	N-Caffeoylputrescine	2.51×10^2	1.63×10^2	2.50×10^2	4.77×10^{-2}	3.67	Up	--	--	--	--	--	--
	pme2244	3-Indolepropionic acid	1.90×10^2	1.18×10^2	1.89×10^2	--	--	--	--	--	--	1.13×10^{-1}	1.21	Up
**Phenolamine**	pmb0490	p-Coumaroylputrescine	2.35×10^2	1.47×10^2	2.34×10^2	1.97×10^{-1}	3.20	Up	--	--	--	--	--	--
	Lmmp001410	Dihydrocaffeoylputrescine	2.53×10^2	1.65×10^2	2.52×10^2	3.79×10^{-1}	5.02	Up	1.88×10^{-1}	6.35	Up	1.35×10^{-1}	5.28	Up
	Lmlp003161	N-Feruloylputrescine	2.65×10^2	1.77×10^2	2.64×10^2	1.35×10^{-1}	1.56	Up	--	--	--	--	--	--
	Lmmp002013	Dihydroferuloylputrescine	2.67×10^2	1.79×10^2	2.66×10^2	9.35×10^{-2}	1.73	Up	--	--	--	--	--	--
	MWSmce089	p-Coumaroyltyramine	2.84×10^2	1.47×10^2	2.83×10^2	1.99×10^{-1}	5.27	Up	--	--	--	--	--	--
	Hmgp102564	Coumaroyl amide derivative	2.95×10^2	2.07×10^2	2.94×10^2	1.37×10^{-1}	1.71	Up	--	--	--	2.69×10^{-2}	1.16	Up

pmb0494	Sinapoylputrescine	2.95×10^{2}	2.07×10^{2}	2.94×10^{2}	1.52×10^{-1}	1.38	Up	--	--	--	--	--	--
pmb0496	N-Feruloylagmatine	3.07×10^{2}	1.77×10^{2}	3.06×10^{2}	1.32×10^{-1}	1.87	Up	3.02×10^{-1}	5.01	Up	2.41×10^{-1}	2.04	Up
mws1433	N-Feruloyltyramine	3.14×10^{2}	1.77×10^{2}	3.13×10^{2}	3.02×10^{-1}	1.29	Up	1.90×10^{-1}	1.06	Up	1.39×10^{-1}	2.40	Up
Hmjp005089	(2Z)-N-[2-(3,4-Dihydroxyphenyl)-2-hydroxyethyl]-3-(4-methoxyphenyl)-2-propenamide	3.30×10^{2}	1.77×10^{2}	3.29×10^{2}	3.82×10^{-1}	5.68	Up	1.45×10^{-2}	1.55	Up	1.66×10^{-1}	1.53	Up
pmb0498	Sinapoylagmatine	3.37×10^{2}	2.07×10^{2}	3.36×10^{2}	2.08×10^{-1}	5.86	Up	2.16×10^{-1}	1.67	Up	9.50×10^{-2}	3.47	Up
HJAP050	N-Feruloyl-3-methoxytyramine	3.44×10^{2}	1.77×10^{2}	3.43×10^{2}	3.65×10^{-1}	3.23	Up	--	--	--	--	--	--
Hmjp006104	N-Cis-Feruloyl-3'-O-methyldopamine	3.44×10^{2}	1.77×10^{2}	3.43×10^{2}	3.63×10^{-1}	3.38	Up	--	--	--	--	--	--
pmb0489	N-Glucosyl-p-coumaroylputrescine	3.97×10^{2}	1.47×10^{2}	3.96×10^{2}	1.94×10^{-1}	8.64	Up	1.92×10^{-2}	-1.63	Down	1.57×10^{-1}	-1.01	Down
pmb0504	N-(4'-O-glycosyl)-feruloyl agmatine	4.69×10^{2}	1.77×10^{2}	4.68×10^{2}	1.11×10^{-1}	2.69	Up	1.14×10^{-1}	2.42	Up	1.85×10^{-2}	3.47	Up
pmp001142	Cimicifugamide A	4.92×10^{2}	1.45×10^{2}	4.91×10^{2}	4.00×10^{-2}	1.06	Up	1.30×10^{-2}	1.96	Up	--	--	--
Hmgp006095	Lyciumamide C	4.92×10^{2}	3.25×10^{2}	4.91×10^{2}	5.61×10^{-2}	5.34	Up	2.08×10^{-1}	1.99	Up	2.15×10^{-1}	4.06	Up
pmb0492	N', N'', N'''-p-Coumaroyl-cinnamoyl-caffeoyl spermidine	5.84×10^{2}	3.25×10^{2}	5.83 × 102	7.65×10^{-2}	1.31	Up	--	--	--	--	--	--
pmn001726	N1-Caffeoyl-N3-dihydrocaffeoylspermidine	4.70×10^{2}	3.34×10^{2}	4.71×10^{2}	--	--	--	1.23×10^{-1}	2.37	Up	9.88×10^{-2}	5.23	Up
Hmgp002804	N1, N10-Dihydrocaffeoylspermidine	4.74×10^{2}	2.22×10^{2}	4.73×10^{2}	--	--	--	1.59×10^{-1}	2.16	Up	7.47×10^{-2}	5.05	Up
Lmmp002914	Bis (Dihydrocaffeoyl)spermidine	4.74×10^{2}	2.22×10^{2}	4.73×10^{2}	--	--	--	1.52×10^{-1}	2.37	Up	6.96×10^{-2}	4.97	Up
pmn001727	N-(4-O-(Glucosyl)-E-feruloyl)-tyramine	4.74×10^{2}	3.12×10^{2}	4.75×10^{2}	--	--	--	3.47×10^{-1}	2.72	Up	--	--	--
pmp001141	Cimicifugamide	5.06×10^{2}	1.45×10^{2}	5.05×10^{2}	--	--	--	5.01×10^{-2}	-1.66	Down	2.98×10^{-1}	-1.07	Down

	Hmgp006701	Lyciumamide A	6.25×10^{2}	3.25×10^{2}	6.24×10^{2}	--	--	--	2.28×10^{-1}	1.75	Up	9.70×10^{-2}	3.94	Up
	MWStz038	Grossamide	6.25×10^{2}	3.25×10^{2}	6.24×10^{2}	--	--	--	2.42×10^{-1}	1.66	Up	1.17×10^{-1}	4.17	Up
	Hmlp000935	Vanillylamine	1.54×10^{2}	1.37×10^{2}	1.53×10^{2}	--	--	--	--	--	--	7.87×10^{-2}	1.48	Up
	Zmzp000616	4-Aminophenol	1.10×10^{2}	93.1	1.09×10^{2}	--	--	--	1.33×10^{-2}	10.68	Up	--	--	--
	Lmqp002784	Salicylamide	1.38×10^{2}	77.0	1.37×10^{2}	--	--	--	2.20×10^{-1}	1.57	Up	--	--	--
	mws4002	Dopamine	1.54×10^{2}	1.37×10^{2}	1.53×10^{2}	--	--	--	--	--	--	4.69×10^{-2}	1.67	Up
	Lmmp001993	Dihydrocaffeoylspermine	3.67×10^{2}	2.22×10^{2}	3.66×10^{2}	--	--	--	--	--	--	5.12×10^{-2}	2.94	Up
	pmp001172	Kukoamine A	5.31×10^{2}	5.13×10^{2}	5.30×10^{2}	--	--	--	--	--	--	5.53×10^{-2}	2.12	Up
	pmn001732	Kukoamine B	5.29×10^{2}	3.65×10^{2}	5.30×10^{2}	--	--	--	--	--	--	1.12×10^{-1}	2.48	Up
	pmp001253	Cannabisin F	6.25×10^{2}	4.88×10^{2}	6.25×10^{2}	--	--	--	--	--	--	1.06×10^{-1}	3.98	Up
	pmp001187	1-(Dihydroxyphenyl)-N2, N3-bis(4-hydroxyphenethyl) -(5-8)-dimethoxy-1,2dihydronaphthalene-2,3-dicarboxamide	6.25×10^{2}	4.60×10^{2}	6.24×10^{2}	--	--	--	--	--	--	1.14×10^{-1}	4.39	Up
	pmp001190	(5-8)-Hydroxy-1-(hydroxylmethoxyphenyl)-N2, N3-bis(4-hydroxyphenethyl)-(5-8)-dimethoxy-1,2-dihydronaphthalene-2,3-dicarboxamide	6.55×10^{2}	5.18×10^{2}	6.54×10^{2}	--	--	--	--	--	--	1.23×10^{-1}	4.18	Up
	Lmmp003591	Tri(dihydrocaffeoyl)spermine	6.95×10^{2}	2.22×10^{2}	6.94×10^{2}	--	--	--	--	--	--	4.32×10^{-2}	1.73	Up
	Hmfp002563	Esculeogenin A	4.48×10^{2}	4.30×10^{2}	4.47×10^{2}	1.49×10^{-2}	2.19	Up	3.24×10^{-2}	1.56	Up	2.56×10^{-3}	2.21	Up
	Hmfp002517	Esculeogenin A-27-O-rhamnoside	5.94×10^{2}	5.76×10^{2}	5.93×10^{2}	7.30×10^{-2}	1.12	Up	4.68×10^{-2}	2.57	Up	9.17×10^{-3}	1.57	Up
**Steroid**	Hmfp002478	Esculeogenin B-O-glucoside	6.10×10^{2}	5.92×10^{2}	6.09×10^{2}	2.86×10^{-2}	1.98	Up	5.80×10^{-2}	1.38	Up	9.68×10^{-3}	2.00	Up

**alkaloids**	Hmfp002048	Esculeogenin A-27-O-glucoside	6.10×10^{2}	5.92×10^{2}	6.09×10^{2}	2.42×10^{-3}	2.00	Up	1.40×10^{-2}	2.02	Up	2.32×10^{-2}	1.54	Up
	Hmfp002251	Esculeogenin B1-O-glucoside	6.10×10^{2}	5.92×10^{2}	6.09×10^{2}	1.00×10^{-3}	1.97	Up	--	--	--	6.88×10^{-2}	2.06	Up
	Hmfp002434	Hydroxytomatidenol-3-O-(6''-acetyl) glucoside	6.34×10^{2}	5.74×10^{2}	6.33×10^{2}	9.22×10^{-2}	1.06	Up	2.89×10^{-3}	1.48	Up	7.79×10^{-3}	2.06	Up
	Hmfp002467	Esculeogenin A-27-O-(6''-acetyl) glucoside	6.52×10^{2}	5.92×10^{2}	6.51×10^{2}	2.32×10^{-4}	1.47	Up	1.12×10^{-2}	2.65	Up	4.90×10^{-2}	4.17	Up
	Hmfp002066	Esculeogenin B-O-sophoroside	7.72×10^{2}	7.54×10^{2}	7.71×10^{2}	5.04×10^{-2}	2.25	Up	--	--	--	--	--	--
	Lmmp004445	Leptinine II	8.85×10^{2}	8.85×10^{2}	8.83×10^{2}	9.64×10^{-2}	1.02	Up	--	--	--	--	--	--
	Lmmp006281	Solasodiene	3.96×10^{2}	3.96×10^{2}	3.95×10^{2}	--	--	--	4.03×10^{-1}	5.20	Up	1.41×10^{-1}	1.75	Up
	Hmmp002720	Solanidine	3.98×10^{2}	3.98×10^{2}	3.97×10^{2}	--	--	--	1.00×10^{-1}	1.92	Up	--	--	--
	Lmmp004621	Leptinidine	4.14×10^{2}	4.14×10^{2}	4.13×10^{2}	--	--	--	1.24×10^{-2}	2.05	Up	1.02×10^{-1}	2.22	Up
	Lmfp004842	Tomatidinol	4.14×10^{2}	2.53×10^{2}	4.13×10^{2}	--	--	--	6.64×10^{-2}	2.22	Up	--	--	--
	Lmfp006712	Soladulcidine	4.16×10^{2}	4.16×10^{2}	4.15×10^{2}	--	--	--	1.40×10^{-1}	2.72	Up	3.42×10^{-1}	4.93	Up
	Hmmp002761	γ-Solanine	5.60×10^{2}	5.60×10^{2}	5.59×10^{2}	--	--	--	1.39×10^{-1}	13.07	Up	--	--	--
	Hmfp003025	Solasodine-3-O-glucoside	5.76×10^{2}	5.58×10^{2}	5.75×10^{2}	--	--	--	8.90×10^{-2}	1.84	Up	2.49×10^{-2}	2.13	Up
	Lmfp004948	δ-Tomatine	5.78×10^{2}	2.55×10^{2}	5.77×10^{2}	--	--	--	9.28×10^{-2}	2.98	Up	1.51×10^{-1}	3.88	Up
	Lmfp004911	γ-Tomatine	7.40×10^{2}	7.40×10^{2}	7.39×10^{2}	--	--	--	6.19×10^{-2}	2.69	Up	1.08×10^{-1}	1.88	Up
	Lmfp004912	β2-Tomatine	8.73×10^{2}	4.16×10^{2}	8.71×10^{2}	--	--	--	8.05×10^{-2}	2.60	Up	1.65×10^{-1}	12.35	Up
	Lmmp004809	β1-Tomatine	9.03×10^{2}	9.03×10^{2}	9.02×10^{2}	--	--	--	1.01×10^{-1}	1.51	Up	8.61×10^{-2}	1.40	Up
	Lmfp005484	Demissine	1.02×10^{3}	1.02×10^{3}	1.02×10^{3}	--	--	--	6.94×10^{-2}	10.73	Up	--	--	--
	Hmfp002852	Tomatidine-3-O-(6''-acetyl) glucosyl-(1→2)-[arabinosyl-(1→3)] glucosyl-(1→4) arabinoside	1.05×10^{3}	1.05×10^{3}	1.05×10^{3}	--	--	--	5.74×10^{-2}	1.29	Up	1.81×10^{-1}	1.22	Up
	Lmfp004629	Dehydrocommersonine	1.05×10^{3}	1.05×10^{3}	1.05×10^{3}	--	--	--	1.37×10^{-2}	1.45	Up	2.68×10^{-1}	1.02	Up
	Hmfp002524	Esculeogenin A-27-O-glucoside-O-	1.05×10^{3}	1.03×10^{3}	1.05×10^{3}	--	--	--	6.97×10^{-2}	1.03	Up	2.13×10^{-3}	1.28	Up

		glucoside-O-Pentoside-O-rhamnoside												
	Lmfp004210	Lycoperoside H	1.05×10^{3}	1.05×10^{3}	1.05×10^{3}	--	--	--	2.11×10^{-2}	2.11	Up	4.87×10^{-3}	1.58	Up
	Hmfp004488	Lycoperoside C	1.09×10^{3}	1.03×10^{3}	1.09×10^{3}	--	--	--	1.05×10^{-1}	4.56	Up	7.46×10^{-2}	2.20	Up
	Lmfp005032	Lycoperoside B	1.09×10^{3}	1.09×10^{3}	1.09×10^{3}	--	--	--	1.62×10^{-1}	3.41	Up	5.41×10^{-3}	3.64	Up
	Lmfp005243	Lycoperoside A	1.09×10^{3}	1.09×10^{3}	1.09×10^{3}	--	--	--	9.52×10^{-2}	4.75	Up	5.44×10^{-2}	2.22	Up
	Hmfp003206	Tomatidine-3-O-glucosyl-(1→2)-[rhamnosyl-(1→3)] glucosyl-(1→4) glucoside	1.05×10^{3}	1.05×10^{3}	1.05×10^{3}	--	--	--	--	--	--	1.01×10^{-1}	1.59	Up
	pmp000149	Cevadine	5.92×10^{2}	5.78×10^{2}	5.91×10^{2}	--	--	--	--	--	--	1.22×10^{-1}	1.98	Up
**Isoquinoline alkaloids**	pmn001692	3-O-Acetylhamayne	3.28×10^{2}	2.95×10^{2}	3.29×10^{2}	2.10×10^{-3}	2.26	Up	1.06×10^{-1}	2.27	Up	1.17×10^{-1}	2.57	Up
	pmp000522	(S)-Canadine	3.40×10^{2}	3.25×10^{2}	3.39×10^{2}	3.57×10^{-1}	3.16	Up	--	--	--	--	--	--
	Cmsp003120	Cephalofortuneine	3.62×10^{2}	1.37×10^{2}	3.61×10^{2}	3.85×10^{-2}	1.80	Up	1.12×10^{-1}	-1.17	Down	--	--	--
**Quinorisidine alkaloids**	mws1108	Sophoridine	2.49×10^{2}	1.50×10^{2}	2.48×10^{2}	4.14×10^{-2}	4.14	Up	--	--	--	--	--	--
**Pyridine alkaloids**	pme1738	3-Carbamyl-1-methylpyridinium;(1-Methylnicotinamide)	1.38×10^{2}	95.0	1.37×10^{2}	--	--	--	--	--	--	4.03×10^{-2}	1.05	Up

Table S3 Differential metabolites of phenolic acids.

Index	Compounds	Q1 (Precursor ion)	Q3 (Fragment ion)	Molecular Weight	WT-GR vs. CR-NOR-like1-GR			WT-BR+3 vs. CR-NOR-like1-BR+3			WT-BR+9 vs. CR-NOR-like1-BR+9		
					***p*-Value**	**Log_2FC**	**Type**	***p*-Value**	**Log_2FC**	**Type**	***p*-Value**	**Log_2FC**	**Type**

MWSmce466	4'-Hydroxyacetophenone	1.35×10^{2}	92.0	1.36×10^{2}	1.22×10^{-1}	-1.12	Down	--	--	--	--	--	--
ML10177402	4-Aminosalicylic acid	1.52×10^{2}	1.08×10^{2}	1.53×10^{2}	1.50×10^{-1}	3.22	Up	5.31×10^{-3}	2.64	Up	1.14×10^{-1}	2.08	Up
Lmrn003000	2-Hydroxy-3-phenylpropanoic acid	1.65×10^{2}	1.03×10^{2}	1.66×10^{2}	9.14×10^{-2}	-2.75	Down	1.33×10^{-1}	-1.40	Down	1.89×10^{-2}	-1.10	Down
Lmbn005172	2,6-Dimethoxybenzaldehyde	1.65×10^{2}	1.19×10^{2}	1.66×10^{2}	9.01×10^{-2}	-2.05	Down	1.69×10^{-1}	-1.24	Down	--	--	--
mws0467	3-(4-Hydroxyphenyl)-propionic acid	1.65×10^{2}	1.19×10^{2}	1.66×10^{2}	7.41×10^{-2}	-1.99	Down	2.03×10^{-1}	-1.11	Down	5.82×10^{-3}	-1.15	Down
pmb2795	4-Methoxycinnamic acid	1.77×10^{2}	1.45×10^{2}	1.78×10^{2}	1.90×10^{-1}	1.34	Up	3.19×10^{-2}	-1.80	Down	5.82×10^{-4}	-1.13	Down
mws1195	p-Coumaric acid methyl ester	1.79×10^{2}	1.47×10^{2}	1.78×10^{2}	1.33×10^{-1}	8.44	Up	1.74×10^{-2}	-1.35	Down	2.03×10^{-2}	-1.35	Down
MWS2070	Propyl 4-hydroxybenzoate	1.79×10^{2}	92.0	1.80×10^{2}	2.54×10^{-1}	-1.64	Down	--	--	--	--	--	--
mws0093	Coniferyl alcohol	1.79×10^{2}	1.46×10^{2}	1.80×10^{2}	8.38×10^{-2}	2.18	Up	--	--	--	9.04×10^{-2}	1.37	Up
pme0422	Isoferulic Acid*	1.93×10^{2}	1.34×10^{2}	1.94×10^{2}	8.00×10^{-2}	1.55	Up	3.84×10^{-2}	2.01	Up	2.13×10^{-2}	2.14	Up
mws0014	Ferulic acid*	1.93×10^{2}	1.34×10^{2}	1.94×10^{2}	6.15×10^{-2}	1.76	Up	1.88×10^{-2}	2.30	Up	1.73×10^{-2}	2.36	Up
Lmdn003756	Methyl caffeate	1.93×10^{2}	1.35×10^{2}	1.94×10^{2}	3.25×10^{-3}	3.38	Up	2.63×10^{-2}	2.75	Up	1.42×10^{-1}	2.59	Up
pme3443	Sinapinaldehyde	2.07×10^{2}	1.92×10^{2}	2.08×10^{2}	2.13×10^{-1}	9.84	Up	--	--	--	--	--	--
Lmhn003240	Benzoylmalic acid	2.37×10^{2}	1.21×10^{2}	2.38×10^{2}	1.88×10^{-1}	1.35	Up	--	--	--	--	--	--
mws1297	Benzyl glucoside	2.69×10^{2}	1.01×10^{2}	2.70×10^{2}	1.14×10^{-1}	1.77	Up	1.16×10^{-2}	2.02	Up	--	--	--
MWSmce675	Arbutin	2.71×10^{2}	1.08×10^{2}	2.72×10^{2}	1.21×10^{-1}	1.01	Up	--	--	--	--	--	--
Hmqp002567	P-Hydroxycinnamic acid p-hydroxyphenethylamine	2.84×10^{2}	1.47×10^{2}	2.83×10^{2}	2.06×10^{-1}	5.64	Up	--	--	--	--	--	--
mws2367	Salidroside	2.99×10^{2}	1.19×10^{2}	3.00×10^{2}	6.08×10^{-2}	1.47	Up	9.73×10^{-3}	1.05	Up	2.47×10^{-2}	1.29	Up
Lmsn002887	1-O-Caffeoyl xylose	3.11×10^{2}	1.63×10^{2}	3.12×10^{2}	1.62×10^{-1}	1.78	Up	--	--	--	--	--	--
Lmtn003441	2-Acetyl-3-hydroxyphenyl-1-O-glucoside	3.11×10^{2}	1.49×10^{2}	3.12×10^{2}	1.37×10^{-1}	2.73	Up	--	--	--	--	--	--

pmn001367	Protocatechuic acid-4-O-glucoside	3.15×10^{2}	1.53×10^{2}	3.16×10^{2}	1.32×10^{-1}	2.19	Up	3.69×10^{-2}	1.24	Up	--	--	--
pmb2871	1-O-Gentisoyl-D-glucoside	3.15×10^{2}	1.53×10^{2}	3.16×10^{2}	1.26×10^{-1}	1.78	Up	--	--	--	--	--	--
Zmtn001661	Vanilloloside	3.15×10^{2}	1.53×10^{2}	3.16×10^{2}	1.39×10^{-1}	2.07	Up	2.69×10^{-2}	1.24	Up	--	--	--
pmb0751	Trans-5-O-(p-Coumaroyl) shikimate	3.21×10^{2}	1.47×10^{2}	3.20×10^{2}	1.75×10^{-1}	1.62	Up	--	--	--	--	--	--
Hmhn003067	Phenylpropionic acid-O-β-D-glucopyranoside	3.25×10^{2}	1.19×10^{2}	3.26×10^{2}	8.18×10^{-2}	-1.27	Down	--	--	--	--	--	--
Lmtn002233	Androsin	3.27×10^{2}	1.65×10^{2}	3.28×10^{2}	1.87×10^{-1}	2.47	Up	3.37×10^{-3}	-1.78	Down	2.86×10^{-2}	-1.05	Down
Zmhn001883	Vanillic acid-4-O-glucoside	3.29×10^{2}	2.09×10^{2}	3.30×10^{2}	3.44×10^{-2}	-1.24	Down	--	--	--	--	--	--
pmb2928	Gallic acid-4-O-glucoside	3.31×10^{2}	3.13×10^{2}	3.32×10^{2}	2.20×10^{-1}	3.99	Up	3.04×10^{-2}	4.43	Up	6.66×10^{-2}	2.60	Up
Lmmn001552	Leonuriside A	3.31×10^{2}	1.69×10^{2}	3.32×10^{2}	9.19×10^{-2}	9.61	Up	--	--	--	6.60×10^{-2}	-1.44	Down
pma6460	4-O-p-Coumaroylquinic acid	3.37×10^{2}	1.63×10^{2}	3.38×10^{2}	2.66×10^{-1}	1.25	Up	1.70×10^{-1}	-1.06	Down	5.24×10^{-2}	-1.17	Down
pmb3074	5-O-p-Coumaroylquinic acid	3.37×10^{2}	1.91×10^{2}	3.38×10^{2}	2.28×10^{-1}	1.42	Up	--	--	--	3.02×10^{-2}	-1.94	Down
pmn001421	3-O-p-Coumaroylquinic acid	3.37×10^{2}	1.91×10^{2}	3.38×10^{2}	2.05×10^{-1}	1.25	Up	--	--	--	2.72×10^{-2}	-1.76	Down
pma0149	Sinapoyl malate	3.41×10^{2}	2.07×10^{2}	3.40×10^{2}	9.57×10^{-2}	1.22	Up	3.21×10^{-2}	1.16	Up	7.79×10^{-2}	1.46	Up
Lmmn000774	Dihydrocaffeoylglucose	3.43×10^{2}	1.81×10^{2}	3.44×10^{2}	2.26×10^{-1}	1.55	Up	4.45×10^{-3}	-1.28	Down	--	--	--
mws0748	1-Caffeoylquinic acid	3.53×10^{2}	1.91×10^{2}	3.54×10^{2}	7.09×10^{-2}	1.39	Up	--	--	--	--	--	--
pmb0752	3-O-Feruloylquinic acid	3.69×10^{2}	1.77×10^{2}	3.68×10^{2}	2.68×10^{-1}	-1.17	Down	1.44×10^{-1}	1.54	Up	--	--	--
pma3724	1-O-Feruloylquinic acid	3.69×10^{2}	1.77×10^{2}	3.68×10^{2}	1.03×10^{-1}	2.16	Up	6.67×10^{-2}	5.04	Up	7.86×10^{-2}	2.03	Up
Lmgn003073	5-O-Feruloylquinic acid	7.35×10^{2}	3.67×10^{2}	3.68×10^{2}	2.04×10^{-1}	4.02	Up	2.43×10^{-1}	9.58	Up	1.64×10^{-1}	3.68	Up
pmn001695	Trihydroxycinnamoylquinic acid	3.71×10^{2}	2.49×10^{2}	3.72×10^{2}	2.26×10^{-2}	1.46	Up	--	--	--	--	--	--
mws0011	Syringin	3.71×10^{2}	2.09×10^{2}	3.72×10^{2}	2.45×10^{-2}	1.29	Up	--	--	--	5.10×10^{-2}	1.44	Up
Cmjn004337	Benzyl-(2''-O-xylosyl) glucoside	4.01×10^{2}	2.69×10^{2}	4.02×10^{2}	5.31×10^{-2}	1.03	Up	3.65×10^{-2}	1.11	Up	1.31×10^{-1}	1.28	Up

Smnn002694	Osmanthuside H [2-(4-Hydroxyphenyl) ethyl-β-D-apiosyl-(1→6)-β-D-glucoside]	4.31×10^{2}	1.49×10^{2}	4.32×10^{2}	9.53×10^{-2}	2.05	Up	5.47×10^{-2}	2.06	Up	7.99×10^{-2}	2.55	Up
pmb3066	5-O-p-Coumaroylshikimic acid O-glucoside	4.81×10^{2}	3.19×10^{2}	4.82×10^{2}	1.64×10^{-2}	10.52	Up	1.41×10^{-2}	-1.79	Down	1.88×10^{-2}	-1.48	Down
Lmgp003989	Dicaffeoylshikimic acid	4.99×10^{2}	1.63×10^{2}	4.98×10^{2}	1.75×10^{-1}	2.54	Up	1.43×10^{-1}	1.33	Up	--	--	--
pmn001384	Isochlorogenic acid C	5.15×10^{2}	3.53×10^{2}	5.16×10^{2}	2.53×10^{-1}	2.27	Up	--	--	--	--	--	--
pmn001710	Rosmarinic acid-3'-O-glucoside	5.21×10^{2}	3.59×10^{2}	5.22×10^{2}	2.58×10^{-1}	1.60	Up	--	--	--	--	--	--
Lmsn003628	6'-O-Sinapoylsucrose	5.47×10^{2}	2.23×10^{2}	5.48×10^{2}	2.13×10^{-2}	1.05	Up	--	--	--	--	--	--
HJN102	3,4,5-Tricaffeoylquinic acid	6.77×10^{2}	5.15×10^{2}	6.78×10^{2}	1.68×10^{-1}	10.56	Up	--	--	--	--	--	--
Lmgp002593	3-hydroxybenzaldehyde	1.23×10^{2}	77.0	1.22×10^{2}	--	--	--	3.24×10^{-2}	-1.14	Down	--	--	--
mws1024	p-Coumaraldehyde	1.47×10^{2}	1.29×10^{2}	1.48×10^{2}	--	--	--	5.15×10^{-2}	-2.98	Down	9.70×10^{-2}	-1.97	Down
pme2362	Mandelic acid	1.51×10^{2}	1.07×10^{2}	1.52×10^{2}	--	--	--	7.72×10^{-2}	-12.43	Down	6.68×10^{-2}	-1.08	Down
mws0639	2,3-Dihydroxybenzoic Acid*	1.53×10^{2}	1.09×10^{2}	1.54×10^{2}	--	--	--	8.21×10^{-3}	-14.50	Down	--	--	--
Lmrn001951	(S)-2-Hydroxy-3-(4-Hydroxyphenyl) Propanoic Acid	1.81×10^{2}	1.35×10^{2}	1.82×10^{2}	--	--	--	6.80×10^{-2}	-1.16	Down	--	--	--
pmb2620	3,4-Dimethoxycinnamic acid	2.07×10^{2}	1.33×10^{2}	2.08×10^{2}	--	--	--	6.75×10^{-2}	1.29	Up	2.51×10^{-2}	1.16	Up
MWSmce083	Ferulic acid methyl ester	2.07×10^{2}	1.33×10^{2}	2.08×10^{2}	--	--	--	2.58×10^{-2}	1.28	Up	--	--	--
mws0853	Sinapyl alcohol	2.09×10^{2}	1.79×10^{2}	2.10×10^{2}	--	--	--	8.12×10^{-2}	-2.35	Down	--	--	--
Lmhn002926	p-Coumaroylmalic acid	2.79×10^{2}	1.63×10^{2}	2.80×10^{2}	--	--	--	1.44×10^{-1}	-1.21	Down	--	--	--
mws1521	Salicin	2.85×10^{2}	1.21×10^{2}	2.86×10^{2}	--	--	--	3.20×10^{-2}	-1.25	Down	--	--	--
Zmhn001358	4-O-Glucosyl-4-hydroxybenzoic acid*	2.99×10^{2}	1.37×10^{2}	3.00×10^{2}	--	--	--	1.26×10^{-2}	-1.29	Down	--	--	--
Lmsn002247	1-O-Salicyloyl-β-D-glucose*	2.99×10^{2}	1.37×10^{2}	3.00×10^{2}	--	--	--	1.83×10^{-2}	-1.16	Down	--	--	--
pmn001553	Cimidahurinine	3.15×10^{2}	1.53×10^{2}	3.16×10^{2}	--	--	--	2.49×10^{-3}	-1.64	Down	2.76×10^{-2}	-1.25	Down

pmb3075	3-O-p-Coumaroylshikimic acid	3.19×10^2	1.45×10^2	3.20×10^2	--	--	--	7.02×10^{-2}	-1.69	Down	--	--	--
Zmzn001549	Ferulic acid-1-O-glucoside	3.25×10^2	1.19×10^2	3.26×10^2	--	--	--	4.52×10^{-4}	-2.41	Down	--	--	--
Lmsn003111	1-O-p-Coumaroyl-β-D-glucose	3.25×10^2	1.45×10^2	3.26×10^2	--	--	--	4.25×10^{-3}	-2.52	Down	--	--	--
Zmhn002301	p-Coumaric acid-4-O-glucoside	3.25×10^2	1.63×10^2	3.26×10^2	--	--	--	3.51×10^{-4}	-2.29	Down	--	--	--
mws1627	Bengenin	3.27×10^2	2.07×10^2	3.28×10^2	--	--	--	6.57×10^{-2}	-1.88	Down	2.76×10^{-2}	-1.25	Down
Hmsn002272	Demethyl coniferin	3.27×10^2	1.65×10^2	3.28×10^2	--	--	--	1.02×10^{-1}	-1.26	Down	8.45×10^{-2}	-1.62	Down
pma0149	Sinapoyl malate	3.41×10^2	2.07×10^2	3.40×10^2	--	--	--	3.21×10^{-2}	1.16	Up	--	--	--
mws0906	Coniferin	3.41×10^2	1.79×10^2	3.42×10^2	--	--	--	1.19×10^{-1}	-1.00	Down	--	--	--
Zmhn002334	6-O-Feruloyl-D-glucose	3.55×10^2	1.93×10^2	3.56×10^2	--	--	--	8.05×10^{-3}	1.44	Up	4.33×10^{-2}	1.16	Up
mws0179	Chlorogenic acid methyl ester	3.67×10^2	1.91×10^2	3.68×10^2	--	--	--	1.13×10^{-1}	3.34	Up	--	--	--
Hmln003529	Benzyl β-primeveroside	4.01×10^2	2.69×10^2	4.02×10^2	--	--	--	1.94×10^{-2}	1.24	Up	--	--	--
Lmyn003028	Benzyl-β-gentiobioside*	4.31×10^2	2.69×10^2	4.32×10^2	--	--	--	1.13×10^{-2}	1.09	Up	--	--	--
Lmtn002324	Benzyl-(2''-O-glucosyl) glucoside*	4.31×10^2	2.69×10^2	4.32×10^2	--	--	--	7.13×10^{-2}	1.29	Up	--	--	--
mad2085	Caffeoyl-p-coumaroyltartaric acid	4.57×10^2	2.95×10^2	4.58×10^2	--	--	--	2.96×10^{-2}	-2.22	Down	--	--	--
Lmhn002321	Vnilloylcaffeoyltartaric acid	4.61×10^2	1.67×10^2	4.62×10^2	--	--	--	1.77×10^{-1}	1.73	Up	3.63×10^{-2}	1.73	Up
Zmhn002750	4-O-(6'-O-Glucosylcaffeoyl)-4-hydroxybenzoic acid	4.61×10^2	3.23×10^2	4.62×10^2	--	--	--	4.57×10^{-2}	-1.20	Down	--	--	--
pmb3064	3-O-p-Coumaroylquinic acid-O-glucoside	4.99×10^2	1.63×10^2	5.00×10^2	--	--	--	3.87×10^{-4}	-1.15	Down	--	--	--
pmb3061	5-O-p-Coumaroylquinic acid O-glucoside	4.99×10^2	1.63×10^2	5.00×10^2	--	--	--	1.04×10^{-2}	-1.04	Down	--	--	--
Lmhn003799	Feruloylferuloyltartaric acid	5.01×10^2	3.07×10^2	5.02×10^2	--	--	--	9.99×10^{-2}	-3.34	Down	--	--	--
pmn001382	Isochlorogenic acid A	5.15×10^2	3.53×10^2	5.16×10^2	--	--	--	1.20×10^{-2}	-14.65	Down	--	--	--
mws2213	Cinnamic acid	1.47×10^2	77.0	1.48×10^2	--	--	--	--	--	--	3.83×10^{-2}	-1.13	Down
Lmsn003318	1-O-Feruloyl-β-D-glucose*	3.55×10^2	1.75×10^2	3.56×10^2	--	--	--	--	--	--	4.75×10^{-3}	1.25	Up

Hmhn003518	4-O-β-D-glucopyranosylferulic acid*	3.55×10^{2}	1.75×10^{2}	3.56×10^{2}	--	--	--	--	--	--	4.53×10^{-3}	1.24	Up
Zmhn002508	4-p-Cumaroyl-rhamnosyl-(1→6)-D-glucose	4.71×10^{2}	3.09×10^{2}	4.72×10^{2}	--	--	--	--	--	--	1.11×10^{-1}	1.77	Up

Table S4 Differential metabolites of flavonoids

**Index**	**Index**	**Compounds**	**Q1 (Precursor ion)**	**Q3 (Fragment ion)**	**Molecular Weight**	**WT-GR vs. CR-NOR-like1-GR**			**WT-BR+3 vs. CR-NOR-like1-BR+3**			**WT-BR+9 vs. CR-NOR-like1-BR+9**		
						***p*-Value**	**Log_2FC**	**Type**	***p*-Value**	**Log_2FC**	**Type**	***p*-Value**	**Log_2FC**	**Type**
**Flavonols**	mws0913	Kaempferol-3-O-galactoside (Trifolin)*	4.47×10^{2}	2.85×10^{2}	4.48×10^{2}	4.13×10^{-2}	-1.35	Down	--	--	--	--	--	--
	MWS20197	Quercetin-3-O-rhamnoside (Quercitrin)	4.49×10^{2}	3.03×10^{2}	4.48×10^{2}	1.77×10^{-1}	1.48	Up	--	--	--	1.56×10^{-2}	1.36	Up
	MWSHY0046	Quercetin-3-O-glucoside (Isoquercitrin)*	4.65×10^{2}	3.03×10^{2}	4.64×10^{2}	5.19×10^{-2}	-1.01	Down	--	--	--	1.24×10^{-1}	-1.15	Down
	Hmcp001769	Quercetin-3-O-rhamnosyl (1→2) arabinoside	5.81×10^{2}	3.03×10^{2}	5.80×10^{2}	3.33×10^{-1}	-1.10	Down	--	--	--	--	--	--
	Lmyp004318	Kaempferol-3-O-(6''-p-Coumaroyl) galactoside	5.95×10^{2}	2.87×10^{2}	5.94×10^{2}	1.60×10^{-1}	2.25	Up	1.59×10^{-1}	1.14	Up	--	--	--
	Lmbp002336	Quercetin-3-O-(2''-O-rhamnosyl) galactoside	6.11×10^{2}	3.03×10^{2}	6.10×10^{2}	2.98×10^{-1}	1.17	Up	--	--	--	1.87×10^{-2}	1.44	Up
	Lmyn001269	Kaempferol-3-O-sophoroside	6.09×10^{2}	2.85×10^{2}	6.10×10^{2}	2.02×10^{-1}	6.25	Up	7.07×10^{-2}	6.62	Up	1.51×10^{-1}	4.55	Up
	Zmcp002666	Quercetin-3,7-Di-O-glucoside	6.27×10^{2}	3.03×10^{2}	6.26×10^{2}	1.87×10^{-1}	1.91	Up	--	--	--	9.60×10^{-2}	1.56	Up

Lmsp003161	Kaempferol-3-O-sophoroside-7-O-rhamnoside	7.57×10^{2}	2.87×10^{2}	7.56×10^{2}	1.17×10^{-1}	1.13	Up	--	--	--	1.86×10^{-2}	1.01	Up
Lmpp003268	Kaempferol-3-O-rutinoside-7-O-glucoside	7.57×10^{2}	2.87×10^{2}	7.56×10^{2}	8.83×10^{-2}	1.35	Up	--	--	--	5.33×10^{-2}	1.63	Up
Hmgp002638	Quercetin-3-O-Soph-7-O-Rha	7.73×10^{2}	4.65×10^{2}	7.72×10^{2}	1.54×10^{-1}	1.37	Up	--	--	--	1.33×10^{-1}	1.20	Up
Lmbp002592	Kaempferol-3,7-di-O-glucoside	6.11×10^{2}	2.87×10^{2}	6.10×10^{2}	--	--	--	1.98×10^{-1}	-1.50	Down	--	--	--
Xmyp004678	Kaempferol-3-O-(2-O-Xylosyl-6-O-Rhamnosyl) Glucoside	7.27×10^{2}	2.87×10^{2}	7.26×10^{2}	--	--	--	3.93×10^{-2}	1.63	Up	--	--	--
Zmhp002640	6-Hydroxykaempferol 3-Rutinoside-6-glucoside	7.73×10^{2}	3.03×10^{2}	7.72×10^{2}	--	--	--	4.65×10^{-2}	-1.20	Down	--	--	--
Lmjp004941	Rhamnocitrin (7-Methylkaempferol)	3.01×10^{2}	2.86×10^{2}	3.00×10^{2}	--	--	--	--	--	--	1.94×10^{-1}	1.16	Up
Zmhp004034	Rhodiolgin	4.65×10^{2}	3.03×10^{2}	4.64×10^{2}	--	--	--	--	--	--	1.26×10^{-2}	1.10	Up
pmn001642	Kaempferol-3-O-(2''-O-acetyl) glucuronide	5.03×10^{2}	4.59×10^{2}	5.04×10^{2}	--	--	--	--	--	--	3.53×10^{-4}	1.68	Up
Lmdp004892	Kaempferol-3-O-(6''-malonyl) galactoside*	5.35×10^{2}	2.87×10^{2}	5.34×10^{2}	--	--	--	--	--	--	1.34×10^{-1}	3.65	Up
Lmmp003817	Kaempferol-3-O-(6''-malonyl) glucoside*	5.35×10^{2}	2.87×10^{2}	5.34×10^{2}	--	--	--	--	--	--	3.04×10^{-2}	2.69	Up
Hmcp001769	Quercetin-3-O-rhamnosy l (1→2) arabinoside	5.81×10^{2}	3.03×10^{2}	5.80×10^{2}	--	--	--	--	--	--	3.47×10^{-2}	1.30	Up

	HJAP148	Kaempferol-3-O-sambubioside	5.81×10^{2}	2.87×10^{2}	5.80×10^{2}	--	--	--	--	--	--	8.90×10^{-2}	1.20	Up
	Lmtp004044	Quercetin-3-O-apiosyl (1 →2) galactoside*	5.97×10^{2}	3.03×10^{2}	5.96×10^{2}	--	--	--	--	--	--	5.64×10^{-2}	1.32	Up
	Lmsp004166	Quercetin-3-O-glucoside-7-O-rhamnoside*	6.11×10^{2}	3.03×10^{2}	6.10×10^{2}	--	--	--	--	--	--	1.10×10^{-3}	1.29	Up
	Zmhp003716	Rhodionidin	6.11×10^{2}	3.03×10^{2}	6.10×10^{2}	--	--	--	--	--	--	5.91×10^{-3}	1.34	Up
	Lmmp002463	Sexangularetin-3-O-glucoside-7-O-rhamnoside	6.25×10^{2}	3.17×10^{2}	6.24×10^{2}	--	--	--	--	--	--	6.38×10^{-3}	1.23	Up
	Lmsp004721	Tamarixetin-3-O-glucoside-7-O-rhamnoside	6.25×10^{2}	3.17×10^{2}	6.24×10^{2}	--	--	--	--	--	--	5.34×10^{-3}	1.07	Up
	HJAP061	Quercetin-3-O-(2''-O-arabinosyl) rutinoside	7.43×10^{2}	3.03×10^{2}	7.42×10^{2}	--	--	--	--	--	--	2.22×10^{-2}	1.24	Up
	Hmcp001618	Quercetin-3-O-(2''-O-Xylosyl) rutinoside	7.43×10^{2}	4.49×10^{2}	7.42×10^{2}	--	--	--	--	--	--	2.90×10^{-2}	1.45	Up
	mws0040	Chrysin	2.55×10^{2}	1.53×10^{2}	2.54×10^{2}	3.45×10^{-1}	12.17	Up	3.77×10^{-1}	13.81	Up	1.13×10^{-1}	8.07	Up
	Hmbp002730	Isoscutellarein	2.87×10^{2}	1.53×10^{2}	2.86×10^{2}	1.47×10^{-1}	10.21	Up	--	--	--	4.46×10^{-3}	1.64	Up
	MWSHY0058	Luteolin (5,7,3',4'-Tetrahydroxyflavone)	2.87×10^{2}	1.53×10^{2}	2.86×10^{2}	1.94×10^{-1}	9.86	Up	--	--	--	1.43×10^{-2}	1.60	Up
**Flavones**	mws0055	Tangeretin (4',5,6,7,8-Pentamethoxyflavone)	3.73×10^{2}	3.43×10^{2}	3.72×10^{2}	2.95×10^{-1}	-1.35	Down	--	--	--	--	--	--
	Hmpp003270	Luteolin-4'-O-glucoside*	4.49×10^{2}	2.87×10^{2}	4.48×10^{2}	2.18×10^{-1}	2.11	Up	--	--	--	3.39×10^{-2}	1.78	Up
	Lmlp003531	Luteolin-3'-O-glucoside*	4.49×10^{2}	2.87×10^{2}	4.48×10^{2}	1.89×10^{-1}	12.83	Up	--	--	--	9.86×10^{-2}	2.24	Up
	pmb2999	Chrysoeriol-5-O-glucoside	4.61×10^{2}	2.99×10^{2}	4.62×10^{2}	3.87×10^{-1}	2.63	Up	--	--	--	--	--	--
	pmb3041	Tricin-7-O-saccharic acid	5.21×10^{2}	3.29×10^{2}	5.22×10^{2}	1.47×10^{-1}	1.86	Up	--	--	--	--	--	--

	pmb3002	Chrysoeriol-7-O-rutinoside	6.07×10^{2}	2.99×10^{2}	6.08×10^{2}	3.23×10^{-2}	-1.03	Down	--	--	--	--	--	--
	pmb3000	Chrysoeriol-7-O-(6''-acetyl) glucoside	5.03×10^{2}	3.41×10^{2}	5.04×10^{2}	--	--	--	9.68×10^{-2}	-1.14	Down	--	--	--
	pmp000593	Luteolin-7-O-rutinoside	5.95×10^{2}	4.49×10^{2}	5.94×10^{2}	--	--	--	6.75×10^{-2}	1.79	Up	--	--	--
	mws0051	Acacetin	2.85×10^{2}	2.70×10^{2}	2.84×10^{2}	--	--	--	--	--	--	3.93×10^{-1}	12.75	Up
	mws4160	Wogonin (5,7-Dihydroxy-8-Methoxyflavone)	2.85×10^{2}	2.70×10^{2}	2.84×10^{2}	--	--	--	--	--	--	3.89×10^{-1}	7.91	Up
	pmb3006	Apigenin-7-O-glucoside (Cosmosiin)	4.31×10^{2}	2.69×10^{2}	4.32×10^{2}	--	--	--	--	--	--	1.72×10^{-1}	1.24	Up
	Lmlp005572	Galangin-7-O-glucoside	4.33×10^{2}	2.71×10^{2}	4.32×10^{2}	--	--	--	--	--	--	7.75×10^{-2}	1.76	Up
	Hmpp002612	Luteolin-7-O-gentiobioside	6.11×10^{2}	2.87×10^{2}	6.10×10^{2}	--	--	--	--	--	--	2.91×10^{-1}	1.22	Up
	Zmxp003107	Luteolin-7,3'-di-O-glucoside	6.11×10^{2}	2.87×10^{2}	6.10×10^{2}	--	--	--	--	--	--	2.29×10^{-1}	1.34	Up
	Hmmp002447	Apigenin-7-O-rutinoside-4'-O-rhamnoside	7.25×10^{2}	4.33×10^{2}	7.24×10^{2}	--	--	--	--	--	--	4.02×10^{-2}	1.50	Up
**Flavanones**	pme3475	Butin	2.73×10^{2}	1.53×10^{2}	2.72×10^{2}	3.05×10^{-1}	8.87	Up	--	--	--	--	--	--
	pme0376	Naringenin (5,7,4'-Trihydroxyflavanone)	2.71×10^{2}	1.51×10^{2}	2.72×10^{2}	3.04×10^{-1}	4.13	Up	--	--	--	--	--	--
	mws0064	Eriodictyol (5,7,3',4'-Tetrahydroxyflavanone)	2.87×10^{2}	1.35×10^{2}	2.88×10^{2}	1.98×10^{-1}	4.50	Up	--	--	--	1.46×10^{-2}	1.51	Up
	Cmxp004977	Isookanin	2.89×10^{2}	1.53×10^{2}	2.88×10^{2}	1.84×10^{-1}	12.59	Up	--	--	--	7.64×10^{-3}	1.90	Up
	Jmgn005927	2-hydroxynaringenin	2.87×10^{2}	1.25×10^{2}	2.88×10^{2}	1.84×10^{-1}	10.23	Up	--	--	--	1.78×10^{-2}	1.48	Up
	mws1033	Homoeriodictyol	3.03×10^{2}	1.53×10^{2}	3.02×10^{2}	2.87×10^{-1}	9.40	Up	1.05×10^{-1}	-2.05	Down	--	--	--
	MWSHY0049	Hesperetin	3.03×10^{2}	1.77×10^{2}	3.02×10^{2}	2.75×10^{-1}	9.49	Up	1.28×10^{-1}	-1.95	Down	--	--	--

	Lmsp004301	3',5,5',7-Tetrahydroxyflavanone-7-O-glucoside	4.51×10^{2}	2.89×10^{2}	4.50×10^{2}	1.24×10^{-1}	2.45	Up	--	--	--	--	--	--
	Jmgn004021	6-C-Glucosyl-2-Hydroxynaringenin	4.49×10^{2}	2.69×10^{2}	4.50×10^{2}	2.10×10^{-1}	1.42	Up	1.01×10^{-2}	1.39	Up	1.69×10^{-1}	1.45	Up
	mws0057	Eriodictyol-7-O-glucoside	4.51×10^{2}	2.89×10^{2}	4.50×10^{2}	1.96×10^{-1}	2.35	Up	--	--	--	--	--	--
	Lmzp002365	Hesperetin-7-O-glucoside	4.65×10^{2}	3.03×10^{2}	4.64×10^{2}	2.41×10^{-2}	1.43	Up	1.98×10^{-1}	1.04	Up	--	--	--
	Cmsp008121	7-O-Methylnaringenin	2.87×10^{2}	1.67×10^{2}	2.86×10^{2}	--	--	--	1.38×10^{-1}	-1.34	Down	--	--	--
	mws1034	Isosakuranetin (5,7-Dihydroxy-4'-methoxyflavanone)	2.87×10^{2}	1.53×10^{2}	2.86×10^{2}	--	--	--	8.30×10^{-2}	-1.08	Down	--	--	--
	Lmqn009304	Eucalyptin (5-Hydroxy-7,4'-dimethoxy-6,8-dimethylflavone)	3.25×10^{2}	2.93×10^{2}	3.26×10^{2}	--	--	--	2.57×10^{-2}	1.38	Up	--	--	--
	Lmyn005006	""Eriodictyol-7-O-(6"""-O-p-coumaroyl) glucoside""	5.95×10^{2}	2.87×10^{2}	5.96×10^{2}	--	--	--	8.40×10^{-2}	-10.63	Down	--	--	--
	Zmhn001036	Choerospondin	4.33×10^{2}	2.71×10^{2}	4.34×10^{2}	--	--	--	--	--	--	2.26×10^{-3}	1.20	Up
**Isoflavones**	Lmmp004504	2'-Hydroxygenistein	2.87×10^{2}	1.53×10^{2}	2.86×10^{2}	2.10×10^{-1}	10.55	Up	--	--	--	7.57×10^{-3}	1.01	Up
	mws0062	Isoluteolin (Orobol)(5,7,3',4'-tetrahydroxyisoflavone)	2.87×10^{2}	2.41×10^{2}	2.86×10^{2}	1.84×10^{-1}	9.13	Up	--	--	--	--	--	--
	Lmdn006025	2-Hydroxy-2,3-dihydrogenistein	2.87×10^{2}	1.25×10^{2}	2.88×10^{2}	1.84×10^{-1}	9.63	Up	--	--	--	1.59×10^{-2}	1.52	Up
	MWSHY0180	Glycitin	4.47×10^{2}	2.85×10^{2}	4.46×10^{2}	2.48×10^{-1}	4.33	Up	6.67×10^{-3}	2.60	Up	3.71×10^{-3}	2.10	Up
	MWSHY0111	Calycosin-7-O-glucoside	4.47×10^{2}	2.85×10^{2}	4.46×10^{2}	2.66×10^{-1}	3.78	Up	1.78×10^{-3}	2.18	Up	3.21×10^{-2}	2.23	Up

	pmp000194	6''-O-Malonylgenistin	5.19×10^{2}	2.71×10^{2}	5.18×10^{2}	--	--	--	1.84×10^{-1}	9.50	Up	5.18×10^{-2}	1.88	Up
	Lmdn007639	3,9-Dihydroxypterocarpan	2.55×10^{2}	1.19×10^{2}	2.56×10^{2}	--	--	--	--	--	--	8.37×10^{-2}	1.32	Up
	Lmmp004504	2'-Hydroxygenistein	2.87×10^{2}	1.53×10^{2}	2.86×10^{2}	--	--	--	--	--	--	7.57×10^{-3}	1.68	Up
	mws0062	Isoluteolin (Orobol)(5,7,3',4'-tetrahydroxyisoflavone)	2.87×10^{2}	2.41×10^{2}	2.86×10^{2}	--	--	--	--	--	--	1.88×10^{-2}	1.51	Up
	Lmgp004731	Genistein-7-O-galactoside	4.33×10^{2}	2.71×10^{2}	4.32×10^{2}	--	--	--	--	--	--	1.54×10^{-1}	1.76	Up
**Chalcones**	pme2960	Naringenin chalcone	2.73×10^{2}	1.53×10^{2}	2.72×10^{2}	2.77×10^{-1}	10.73	Up	--	--	--	--	--	--
	pme1201	Phloretin	2.73×10^{2}	1.67×10^{2}	2.74×10^{2}	3.69×10^{-1}	12.18	Up	--	--	--	--	--	--
	Cmxp005429	Okanin	2.89×10^{2}	1.53×10^{2}	2.88×10^{2}	1.98×10^{-1}	4.92	Up	--	--	--	4.23×10^{-3}	1.86	Up
	mws2118	Phloretin-2'-O-glucoside (Phlorizin)	4.35×10^{2}	1.67×10^{2}	4.36×10^{2}	2.95×10^{-2}	-9.23	Down	--	--	--	--	--	--
	Hmpn005101	Sieboldin	4.51×10^{2}	2.89×10^{2}	4.52×10^{2}	1.53×10^{-1}	1.42	Up	1.32×10^{-2}	1.49	Up	9.59×10^{-2}	1.39	Up
	Cmxn004016	Dihydromarein	4.51×10^{2}	2.89×10^{2}	4.52×10^{2}	1.79×10^{-1}	1.32	Up	2.22×10^{-4}	1.26	Up	4.13×10^{-2}	2.08	Up
	pme3217	Isoliquiritigenin	2.57×10^{2}	1.37×10^{2}	2.56×10^{2}	--	--	--	--	--	--	1.23×10^{-1}	1.22	Up
	Lmmp007480	2,4,4'-trihydroxychalcone	2.57×10^{2}	1.37×10^{2}	2.56×10^{2}	--	--	--	--	--	--	1.75×10^{-1}	1.12	Up
	Lmsn003297	3,4,2',4',6'-Pentahydroxychalcone-4'-O-glucoside	4.49×10^{2}	2.87×10^{2}	4.50×10^{2}	--	--	--	--	--	--	7.27×10^{-2}	2.68	Up
	Lmsn006753	Phloretin-4'-O-(6''-p-Coumaroyl) glucoside	5.81×10^{2}	2.73×10^{2}	5.82×10^{2}	--	--	--	--	--	--	1.69×10^{-1}	1.27	Up
**Flavanonols**	mws0914	Pinobanksin	2.71×10^{2}	1.51×10^{2}	2.72×10^{2}	2.98×10^{-1}	5.39	Up	--	--	--	--	--	--
	mws1094	Aromadendrin (Dihydrokaempferol)	2.87×10^{2}	2.59×10^{2}	2.88×10^{2}	2.19×10^{-1}	7.40	Up	--	--	--	--	--	--
	mws1360	Engeletin	4.33×10^{2}	2.69×10^{2}	4.34×10^{2}	1.86×10^{-1}	-9.22	Down	--	--	--	--	--	--

	Lmtn002796	Aromadendrin-7-O-glucoside	4.49×10^{2}	2.87×10^{2}	4.50×10^{2}	--	--	--	1.79×10^{-2}	-1.08	Down	--	--	--
	Lmmn004625	Dihydrokaempferol-7-O-glucoside	4.49×10^{2}	2.87×10^{2}	4.50×10^{2}	--	--	--	1.05×10^{-1}	1.50	Up	--	--	--
	mws0044	Dihydroquercetin (Taxifolin)	3.03×10^{2}	1.25×10^{2}	3.04×10^{2}	--	--	--	--	--	--	1.30×10^{-1}	1.08	Up
	pmp000127	Chrysoeriol-6,8-di-C-glucoside	6.25×10^{2}	6.07×10^{2}	6.24×10^{2}	7.35×10^{-2}	1.96	Up	2.24×10^{-2}	2.66	Up	6.62×10^{-2}	2.72	Up
**Flavonoid carbonosides**	pmb0623	Chrysoeriol-6-C-glucoside-7-O-glucoside	6.25×10^{2}	6.07×10^{2}	6.24×10^{2}	1.04×10^{-1}	1.96	Up	1.70×10^{-2}	3.20	Up	1.03×10^{-1}	2.81	Up
	Lmnp002413	Luteolin-6-C-rhamnoside-7-O-glucoside	5.95×10^{2}	4.49×10^{2}	5.94×10^{2}	--	--	--	4.69×10^{-2}	1.70	Up	--	--	--
	pmb0639	Apigenin-8-C-glucoside-7-O-Sophoroside	7.57×10^{2}	5.95×10^{2}	7.56×10^{2}	--	--	--	1.85×10^{-1}	-1.41	Down	--	--	--
**Flavanols**	Lmmp000897	Gallocatechin-(4α→8)-gallocatechin	6.11×10^{2}	2.87×10^{2}	6.10×10^{2}	--	--	--	9.41×10^{-2}	-1.65	Down	--	--	--
	mws0355	Catechin gallate*	4.41×10^{2}	1.69×10^{2}	4.42×10^{2}	--	--	--	--	--	--	2.00×10^{-1}	-1.78	Down
**Aurones**	pmp000964	Aureusidin	2.87×10^{2}	1.53×10^{2}	2.86×10^{2}	1.84×10^{-1}	10.20	Up	--	--	--	7.10×10^{-3}	1.64	Up
**Other Flavonoids**	pmp001033	Isomangiferin	4.23×10^{2}	4.05×10^{2}	4.22×10^{2}	--	--	--	2.88×10^{-2}	1.39	Up	--	--	--
